# The value of right ventricular to pulmonary arterial coupling in the critically ill: a National Echocardiography Database of Australia (NEDA) substudy

**DOI:** 10.1186/s13613-024-01242-0

**Published:** 2024-01-16

**Authors:** Emma Bowcock, Stephen Huang, Rachel Yeo, Deshani Walisundara, Chris F. Duncan, Faraz Pathan, Geoffrey Strange, David Playford, Sam Orde

**Affiliations:** 1 Intensive Care Medicine, Nepean Hospital, Sydney, Australia; 2 University of Sydney, Sydney, Australia; 3 Intensive Care Medicine, John Hunter Hospital, Newcastle, Australia; 4 Department of Cardiology, Nepean Hospital, Sydney, Australia; 5 The University of Notre Dame, Fremantle, Australia

**Keywords:** TAPSE, Pulmonary hypertension, Echocardiography, Ventriculo-arterial coupling, Ventricular dysfunction, Critical illness, Intensive care units, Survival

## Abstract

**Background:**

Right ventricular (RV) function is tightly coupled to afterload, yet echocardiographic indices of RV function are frequently assessed in isolation. Normalizing RV function for afterload (RV-PA coupling) using a simplified ratio of tricuspid annular plane systolic excursion (TAPSE)/ tricuspid regurgitant velocity (TRV) could help to identify RV decompensation and improve risk stratification in critically ill patients. This is the first study to explore the distribution of TAPSE/TRV ratio and its prognostic relevance in a large general critical care cohort.

**Methods:**

We undertook retrospective analysis of echocardiographic, clinical, and mortality data of intensive care unit (ICU) patients between January 2012 and May 2017. A total of 1077 patients were included and stratified into tertile groups based on TAPSE/TRV ratio: low (< 5.9 mm.(m/s)^−1^), middle (≥ 5.9–8.02 mm.(m/s)^−1^), and high (≥ 8.03 mm.(m/s)^−1^). The distribution of the TAPSE/TRV ratio across ventricular function subtypes of normal, isolated left ventricular (LV), isolated RV, and biventricular dysfunction was explored. The overall prognostic relevance of the TAPSE/TRV ratio was tested, including distribution across septic, cardiovascular, respiratory, and neurological subgroups.

**Results:**

Higher proportions of ventricular dysfunctions were seen in low TAPSE/TRV tertiles. TAPSE/TRV ratio is impacted by LV systolic function but to a lesser extent than RV dysfunction or biventricular dysfunction. There was a strong inverse relationship between TAPSE/TRV ratio and survival. After multivariate analysis, higher TAPSE/TRV ratios (indicating better RV-PA coupling) were independently associated with lower risk of death in ICU (HR 0.927 [0.872–0.985], *p* < 0.05). Kaplan–Meier analysis demonstrated higher overall survival in middle and high tertiles compared to low tertiles (log rank *p* < 0.0001). The prognostic relevance of TAPSE/TRV ratio was strongest in respiratory and sepsis subgroups. Patients with TAPSE/TRV < 5.9 mm (m/s)^−1^ had a significantly worse prognosis than those with higher TAPSE/TRV ratios.

**Conclusion:**

The TAPSE/TRV ratio has prognostic relevance in critically ill patients. The prognostic power may be stronger in respiratory and septic subgroups. Larger prospective studies are needed to investigate the role of TAPSE/TRV in pre-specified subgroups including its role in clinical decision-making.

**Supplementary Information:**

The online version contains supplementary material available at 10.1186/s13613-024-01242-0.

## Background

Right ventricular (RV) dysfunction is common in critically ill patients, occurring frequently in the context of increased right ventricular afterload, and often exacerbated by mechanical ventilation [1–3]. It remains challenging to identify higher risk subgroups with RV dysfunction that have developed, or are at risk of developing, RV decompensation, which progresses to RV failure without timely intervention [4–6]. An early hallmark of RV decompensation or maladaptation is the point of RV ‘uncoupling’ from the pulmonary arterial circulation (RV–PA uncoupling) [4, 5, 7].

RV–PA coupling refers to effective energy transfer from the RV to the pulmonary circulation. When coupling is optimal, flow occurs at the lowest energy expenditure [7]. RV-PA coupling is challenged in the setting of either RV systolic impairment or increased RV afterload, thus treatment strategies may focus on reducing RV afterload and/or improving contractility. Multi-beat pressure–volume (PV) loop analysis is the gold standard method of assessment of RV-PA coupling using the ratio of right ventricular elastance (Ees) to effective pulmonary arterial elastance (Ea), Ees/Ea [8, 9]. Normal values for the Ees/Ea ratio (RV-PA coupling) are between 1.5 and 2 [7, 10]. Uncoupling with maladapative RV responses occur at Ees/Ea ratios of around 0.7, indicating considerable reserve in non-critically unwell cohorts [10, 11]. Unfortunately, no such blueprint data exists in the critically ill, and invasive methods remain impractical at the bedside[12, 13]. Consequently, non-invasive RV–PA coupling surrogates have been investigated [14–16]. First proposed by Guazzi et al., echocardiographic assessment of tricuspid annular plane systolic excursion (TAPSE) versus pulmonary artery systolic pressure (PASP) can be taken as an index of changes in RV length (TAPSE) versus developed force (PASP) and provides a non-invasive measure of RV contractile state and RV-PA coupling beyond the information provided by each separate variable [17]. TAPSE/PASP ratio has emerged as a superior metric to discriminate RV-PA uncoupling in pulmonary hypertension (PH) and has strong independent prognostic relevance in PH and heart failure groups [15, 18, 19]. Prognostic signals are also found in critically ill patients with sepsis, acute respiratory distress syndrome (ARDS) [20, 21] and in patients with acute pulmonary embolism (PE) [22]. PASP measurement requires *accurate* assessment of right atrial pressure (RAP), which is difficult to achieve using echocardiography in the critically ill [23]. In a recent study the TAPSE/tricuspid regurgitation maximal velocity (TRV) ratio emerged as a powerful prognostic coupling metric with a ratio of $\le$ 3.74 mm (m/s)^−1^ improving risk stratification in PH cohorts above TAPSE alone [24]. We, therefore, sought to explore this simplified coupling metric, using TRV as a surrogate for RV afterload and using TAPSE/TRV instead of TAPSE/PASP [24] (Additional file 1: Figure S1).

First, because TAPSE and RV–PA coupling metrics are impacted by left ventricular (LV) function [25, 26], we evaluated the influence of ventricular function on the TAPSE/TRV ratio. In addition, there are no data on TAPSE/TRV across different diagnostic subgroups in critical illness; therefore, we compared the distribution of TAPSE/TRV across a range of diagnostic subgroups. Second, this study aimed to assess whether TAPSE/TRV ratio has prognostic relevance in critically ill patients.

## Methods

**Fig. 1:**
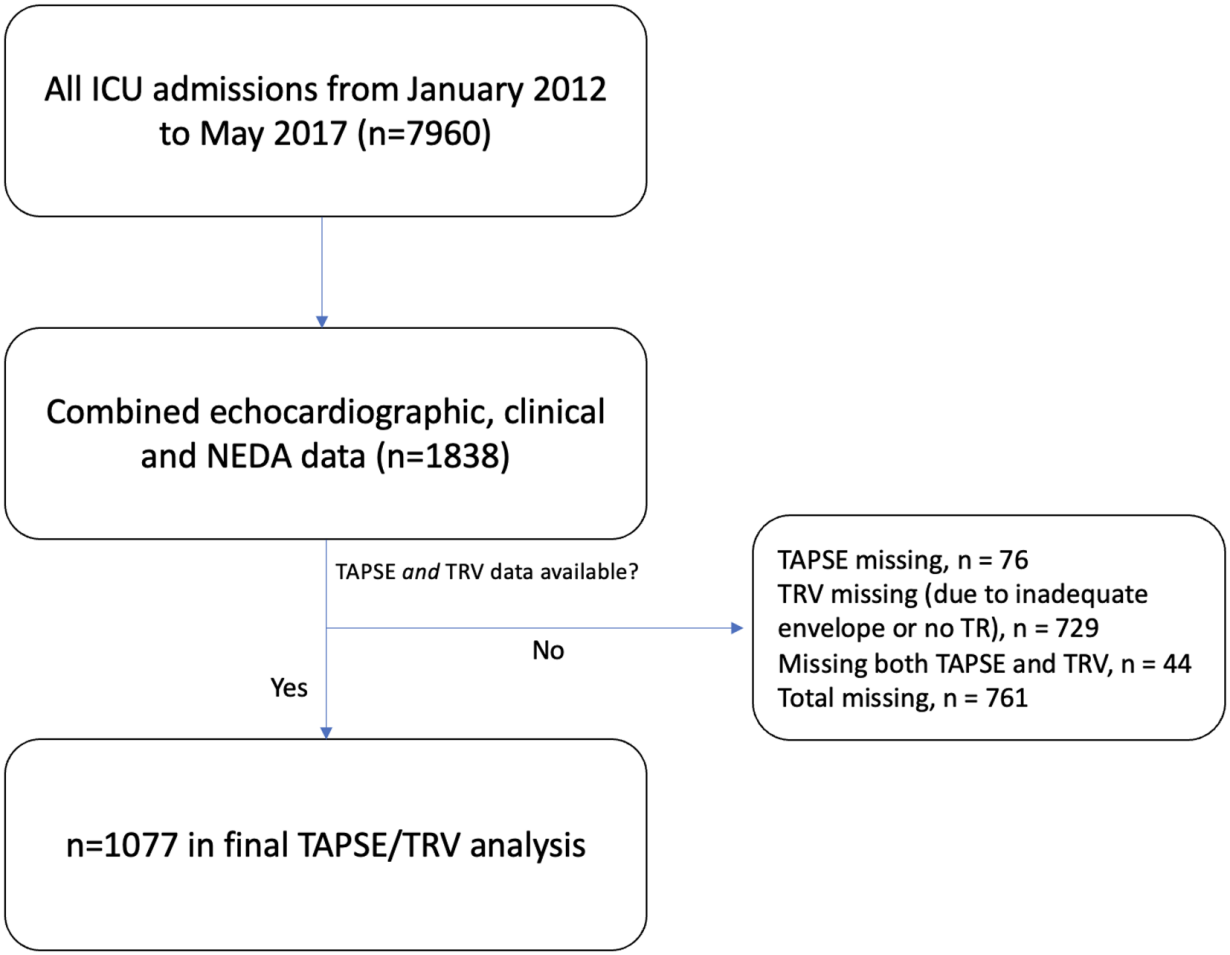
Study Flow Chart. *TRV* tricuspid regurgitation maximal velocity; *TAPSE* tricuspid annular plane systolic excursion; *NEDA* national echocardiographic database of Australia; *ICU* intensive care unit

Echocardiographic data from Nepean Hospital, Sydney, Australia provided to the National Echocardiographic Database of Australia (NEDA) were merged with data from the Intensive Care Unit (ICU). The NEDA study is a large observational database of echo data and is registered with the Australian New Zealand Clinical Trials Registry (ACTRN12617001387314) [27]. It has approval from all relevant Human Research Ethics Committees and adheres to the Declaration of Helsinki. The study cohort included all patients from Nepean ICU that had a clinical indication for transthoracic echocardiogram (TTE) during ICU admission between January 2012 and May 2017. Clinical and health outcome data for the patients were derived from the core outcome measurement and evaluation tool (COMET) and local echo databases. All data were combined and transferred into a central database. Individuals over the age of 18 years were included. Long-term survival data were extracted from NEDA census data obtained on the 21/05/2019. This data was used to define long-term outcome. The range of long-term follow up was a minimum 2 years (for those admitted in May 2017) and maximum 7.5 years (for those admitted in January 2012). Reports with missing TAPSE or TRV data were identified (DW, RY, CD and EB). Of 7960 patients reviewed, 1838 patients had combined clinical, echo and NEDA mortality data with 761 patients having missing data for TAPSE and/or TRV (Fig. 1). All data were then cleaned and transformed to generate echo profiling data and to remove incomplete measurements. A total of 1077 patients were included in final analysis.

The four APACHE diagnostic categories most frequently receiving a TTE during ICU admission were selected for subgroup analysis (respiratory, cardiovascular, sepsis and neurological). Four ventricular function subgroups were pre-defined: normal, isolated left ventricle (LV), isolated RV and biventricular dysfunction. All echocardiography reports were performed by experienced clinicians with advanced echocardiographic qualification. LV dysfunction was defined as an ejection fraction (EF) of < 50%; this value was previously used as a cut off to denote reduced LV systolic function when assessing RV–PA coupling [17]. Of the 1077 patients with TAPSE/TRV data only 384 (36%) had Simpson’s biplane EF, for the remainder, EF was estimated by visual assessment. RV dysfunction was defined by a TAPSE < 17 mm. Biventricular dysfunction was defined as LVEF < 50% *and* TAPSE < 17 mm. Degrees of RA and RV enlargement, obtained from the apical 4 chamber view, were reported as normal, mild, moderate, or severe, based on visual assessment [14]. Degrees of valvular regurgitation severity were reported as mild, moderate, or severe based on local echocardiography laboratory protocols that follow American Society of Echocardiography (ASE) guidelines [28].

TRV was measured from continuous wave Doppler peak velocity and TAPSE from a standard apical four chamber view using M-Mode. RV–PA coupling was assessed using TAPSE/TRV ratios, with higher TAPSE/TRV reflecting better RV–PA coupling (14). Patients were divided into subgroups based on TAPSE/TRV ratio tertiles [14] as follows: low (< 5.9 mm.(m/s)^−1^), middle (≥ 5.9–8.02 mm.(m/s)^−1^), and high (≥ 8.03 mm.(m/s)^−1^) **(**Additional file 1: Figure S2).

### Statistical analysis

NEDA data analyses and reports conform to the STROBE and RECORD guidelines where possible [29, 30]. Continuous data were expressed as median and interquartile range [IQR]; categorical variables were expressed as counts and percentages. For comparisons between continuous variables a two-sided independent t-test and Mann–Whitney *U* test were used for normally distributed and non-normally distributed variables, respectively. Between-group differences were analyzed with one-way ANOVA, Kruskal–Wallis test, Pearson Chi-square test or Wilcoxon-rank method as appropriate. Normality was tested using Shapiro–Wilk test. Univariate and multivariate Cox proportional hazard regression prediction models were used to assess the prognostic relevance of TAPSE/TRV ratios. In multivariate analysis, covariate selection was based on clinical relevance and included TAPSE/TRV as a continuous variable alongside age, sex, APACHE 3 score, invasive ventilation, chronic cardiovascular disease, chronic respiratory disease, and LV systolic function. TAPSE was not included to avoid overfitting with collinearity. The corresponding survival curves were calculated by the Kaplan–Meier method and p values derived with the log-rank test. All statistical tests were two-sided, and a *p*-value of < 0.05 was considered statistically significant. Statistical analyses were performed using Jamovi software 2022, version 2.3 and R (version 4.2.0, R Foundation for Statistical Computing, Vienna, Austria, https://www.R-project.org/).

## Results

### Study population.

1077 adult patients were included in our study with TAPSE/TRV values for analysis with 359 patients in each tertile. Overall ICU survival and long-term survival (survival at time point 21/05/2019) was 84.2% and 42.5%, respectively. Overall, the median age was 69 years [58, 77], and 47% of patients were male. 49.8% received invasive mechanical ventilation. No significant difference in TAPSE/TRV ratio was found between those who did and did not receive invasive ventilation (6.9 [5.5, 8.8] and 6.9 [5.3, 8.8], *p* = 0.43, respectively). Median ICU length of stay was 3.7 [2.0, 7.3] days and hospital length of stay 11.7 [6.1, 23] days. Median time to TTE from ICU admission was 2 [1, 3] days.

Table 1 summarizes patient characteristics and pre-existing comorbidity across TAPSE/TRV tertile groups. Patients in the low TAPSE/TRV tertile were older (*p* < 0.001) and had significantly more chronic cardiovascular (*p* < 0.001), chronic respiratory (*p* = 0.03) and chronic renal disease (p = 0.05). Diagnostic categories receiving TTE are shown in Additional file 1: Figure S3.

**Table 1:** Patient characteristics across TAPSE/TRV tertiles

TAPSE/TRV tertiles			
Characteristic	Low (*N* = 359)	Middle (*N* = 359)	High (*N* = 359)
TAPSE/TRV ratio	4.73 (3.81, 5.38)	6.92 (6.44, 7.46)	9.78 (8.83, 11.04)
Age (years)	73 (64, 79)	70 (60, 78)	63 (52, 73)
Sex			
Female	189 (53%)	191 (53%)	191 (53%)
Male	170 (47%)	168 (47%)	168 (47%)
APACHE 3 score	72 (58, 88)	65 (51, 86)	62 (46, 80)
(Missing)	12	11	5
Medical/Surgical			
Medical	322 (90%)	314 (87%)	298 (83%)
Surgical	37 (10%)	45 (13%)	61 (17%)
Invasive ventilation			
Not ventilated	187 (52%)	180 (50%)	174 (48%)
Ventilated	172 (48%)	179 (50%)	185 (52%)
APACHE comorbidities			
Cirrhosis	10 (2.8%)	12 (3.3%)	13 (3.6%)
Chronic respiratory disease	62 (17%)	48 (13%)	38 (11%)
Chronic cardiovascular disease	45 (13%)	26 (7.2%)	5 (1.4%)
Chronic renal disease	18 (5.0%)	18 (5.0%)	7 (1.9%)
Immunosuppressed	57 (16%)	64 (18%)	47 (13%)
Hepatic failure	2 (0.6%)	1 (0.3%)	0 (0%)
Metastatic cancer	9 (2.5%)	13 (3.6%)	13 (3.6%)
ICU length of stay (days)	3.7 (2.1, 7.2)	3.8 (2.1, 7.0)	3.8 (1.9, 7.9)
ICU outcome			
Died	71 (20%)	58 (16%)	41 (11%)
Survived	288 (80%)	301 (84%)	318 (89%)
Hospital length of stay (days)	12 (6, 22)	11 (6, 22)	11 (6, 27)
(Missing)	2	6	1
Hospital outcome			
Died	109 (30%)	83 (23%)	59 (16%)
Survived	249 (70%)	274 (77%)	300 (84%)
(Missing)	1	2	0
Long-term outcome			
Dead	253 (70%)	207 (58%)	159 (44%)
Survived	106 (30%)	152 (42%)	200 (56%)

Baseline characteristics, comorbidities, length of stay and survival according to TAPSE/TRV tertile. Median (IQR), *n* (%)

### Relationship of TAPSE/TRV tertiles to echocardiographic parameters

Echocardiographic characteristics across TAPSE/TRV tertiles are shown in Additional file 1: Table S1. LVEF (by Simpsons Biplane) was higher in the high versus low tertile group (55% [45, 65] vs. 35% [25, 49], *p* < 0.001, respectively) and was positively correlated with TAPSE/TRV (*r* 0.458, *p* < 0.001). With regards to surrogates of LV diastolic function, there was no significant difference in E/A ratio across tertiles, however, the E/e’ ratio (a surrogate for LA pressure) was higher in the low tertile versus high tertile group (10.0 [9.1, 13.5] vs. 9.2 [7.1, 10.4], *p* < 0.001, respectively). The low tertile group had larger left atrial size when compared to the high tertile group (4.0 cm [3.5, 4.6] vs. 3.6 cm [3.2, 3.9]), *p* < 0.001, respectively).

Patients with moderate/severe dilatation of the RV and right atrium (RA) had significantly lower TAPSE/TRV ratios compared to patients with no/mild enlargement (TAPSE/TRV ratio 4.88 vs. 7.26 and TAPSE/TRV 5.07 vs. 7.38, *p* < 0.0001, respectively) **(**Additional file 1: Figure S4 c and d, respectively). TAPSE/TRV ratios were significantly lower in those with moderate or severe tricuspid regurgitation (TR) than in patients with trace/mild TR (TAPSE/TRV ratio 5.18 vs. 6.95, *p* < 0.001, respectively) (Additional file 1: Figure S4b). The same was also true for mitral regurgitation (Additional file 1: Figure S4a).

### TAPSE/TRV ratio distribution across diagnostic and ventricular dysfunction subgroups

Of the 1077 patients, 644 (60%) had normal ventricular function, 143 (13%) had isolated LV dysfunction, 123 (11%) had isolated RV dysfunction and 167 (16%) had biventricular dysfunction. The corresponding median (IQR) TAPSE/TRV values across ventricular subgroups were: 7.95 [6.59, 9.74] for normal, 4.26 [3.56, 5.51] for biventricular dysfunction, 7.08 [6.04, 8.40] for LV dysfunction and 4.66 [3.79, 5.47] for RV dysfunction. The relationship between TAPSE/TRV tertiles across ventricular dysfunction patterns stratified by diagnostic subgroups are shown in Additional file 1: Figure S**5.** Across all diagnostic subgroups, lower TAPSE/TRV ratios were found in those with RV and biventricular dysfunction as compared to no or isolated LV dysfunction **(**Additional file 1: Figure S5).

TAPSE/TRV tertiles were stratified across four diagnostic subgroups: cardiovascular (*n* = 217), respiratory (*n* = 335), neurological (*n* = 110) and sepsis (*n* = 176). Additional file 1: Table S2 shows patient characteristics and TAPSE/TRV ratios across diagnostic subgroups. Overall Kaplan–Meier survival curves and Cox hazard regression tables across diagnostic subgroups are shown in Additional file 1: Figure S6. Higher survival was found in neurological and respiratory subgroups compared to cardiovascular and sepsis subgroups (HR = 0.68 [0.54–0.86), *p* = 0.001 and HR = 0.72 [0.61–0.86), *p* < 0.001, respectively).

**Fig. 2:**
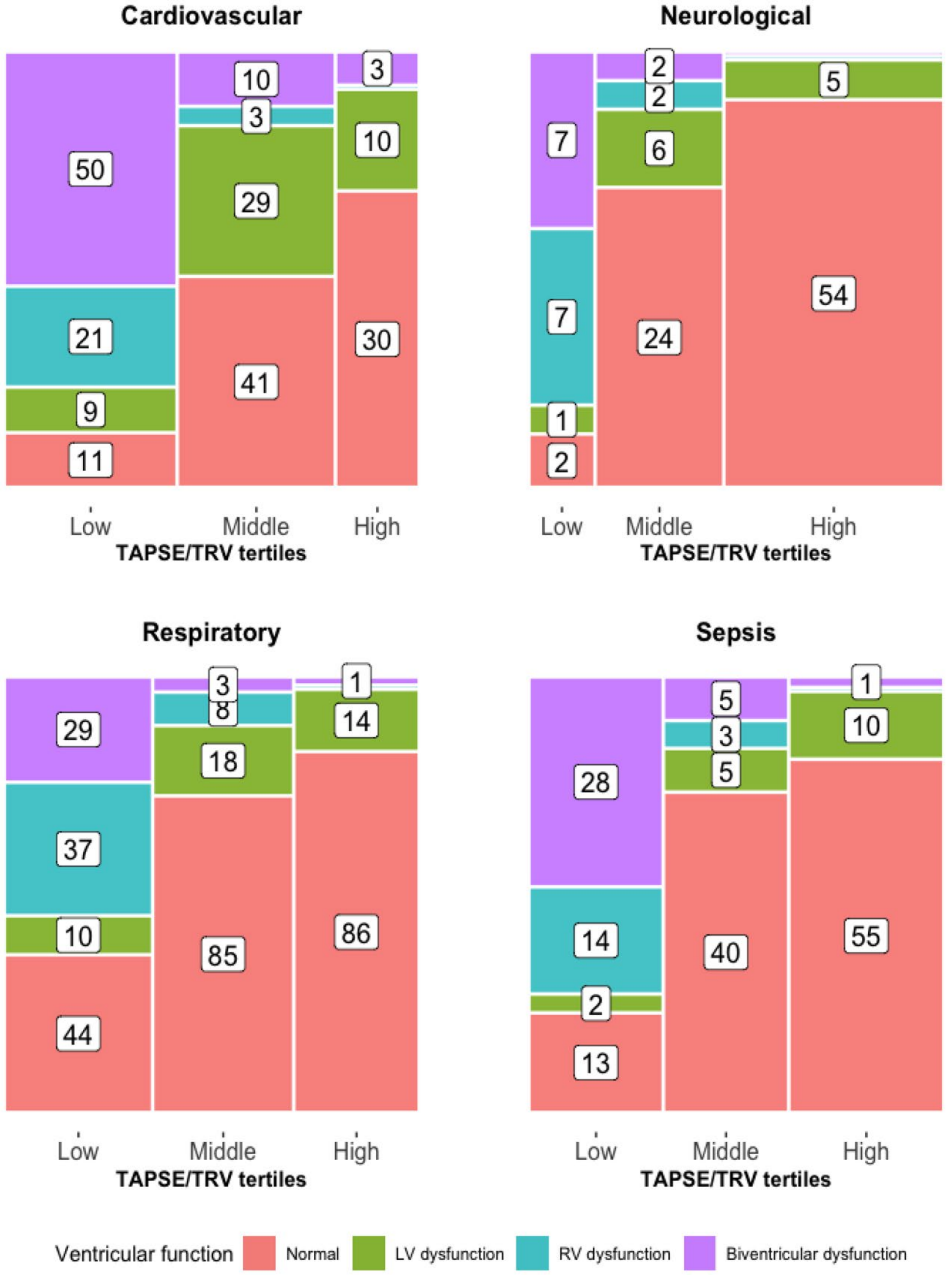
TAPSE/TRV tertiles and ventricular dysfunction subgroups stratified across diagnostic categories. The width x height (i.e., area) of the columns represents the proportion within each tertile and color coding represents the proportion of corresponding ventricular dysfunction patterns within TAPSE/TRV tertiles. Total number of patients with TAPSE/TRV values in each diagnostic subgroup: cardiovascular (*n* = 217), respiratory (*n* = 335), neurological (*n* = 110) and sepsis (*n* = 176)

As displayed in Fig. 2, the diagnostic subgroups displayed different proportions of TAPSE/TRV ratios and ventricular dysfunction patterns. In respiratory subgroups, isolated RV dysfunction was predominant in the low tertile. However, forty-four (36%) patients in the respiratory subgroup were in the low TAPSE/TRV tertile despite normal ventricular function. The cardiovascular subgroup had the highest proportion of low TAPSE/TRV ratios, and biventricular dysfunction was predominant in the low tertile. A greater proportion of patients with sepsis had high TAPSE/TRV ratios and the majority of these had normal ventricular function; biventricular dysfunction and RV dysfunction subtypes were predominant in the low TAPSE/TRV tertile. The high TAPSE/TRV tertile with normal ventricular function was predominant in patients with neurological diagnoses. Overall, patients in low and middle tertiles had higher rates of ventricular dysfunctions when compared to the high tertile.

**Fig. 3:**
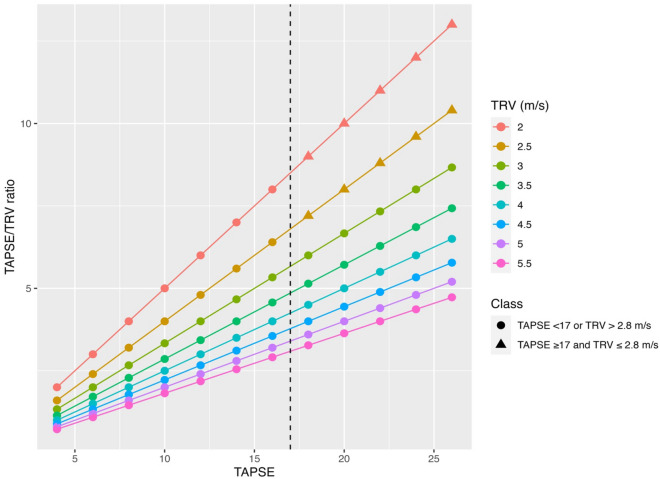
Relationship of TAPSE, TRV and TAPSE/TRV ratio in 1077 patients. As can be seen, some patients had a TAPSE < 17mm and low TRV, or TAPSE > 17mm and a high TRV, giving higher, *pseudo-normalised* TAPSE/TRV ratios. Fewer patients had TAPSE ≥ 17 mm and TRV ≤ 2.8 m/s. A TRV of > 2.8 m/s is used as a cut off to increase probability of pulmonary hypertension [34]

The relationship between TAPSE, TRV, and TAPSE/TRV is shown in Fig. 3. A proportion of patients had reduced TAPSE (< 17mm), normal TRV ($\le 2.8m/s)$ with preserved TAPSE/TRV ratio, or a high TAPSE, high TRV and preserved TAPSE/TRV ratio, representing *pseudo-normalised* TAPSE/TRV groups. Additional file 1: Figure S7 shows the numbers of patients in each ‘TAPSE/TRV ratio-TAPSE-TRV’ category across the four diagnostic subgroups. The fractions represent the number with increased TRV (> 2.8 m/s) within that category. If a TAPSE/TRV ratio of 8.03 is used as the normal cut point, 11 patients were in the upper left panel with a reduced TAPSE, normal TRV and normal TAPSE/TRV ratio, representing a *pseudo-normal TAPSE/TRV group with a reduced TAPSE.* There were 51 patients in the upper right panel with a high TAPSE, high TRV and normal TAPSE/TRV ratio, representing a *pseudo-normal TAPSE/TRV group with an increased TRV.* Respiratory, cardiovascular, and sepsis subgroups had significant proportions of pseudo-normal TAPSE/TRV ratio with an increased TRV (24/99 (24%), 8/38 (21%) and 12/64 (19%), respectively), whereas the proportion with pseudo-normal ratios in neurological subgroups was lower (7/57 (12%)).

**Fig. 4:**
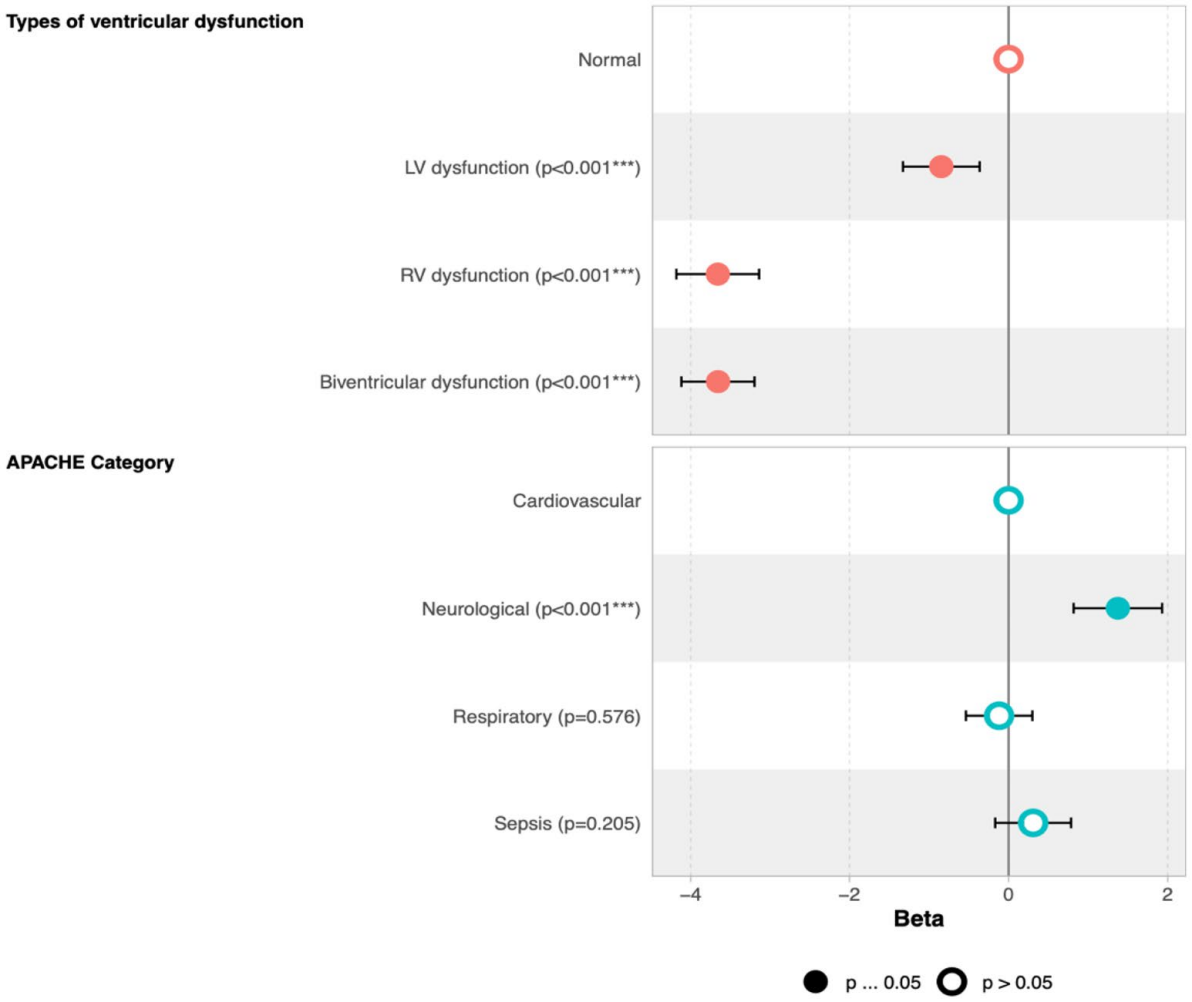
Multivariate analysis of effects of ventricular function and diagnostic categories on TAPSE/TRV ratio. Top: Keeping diagnostic category constant, the TAPSE/TRV ratios were significantly lower in LV, RV and biventricular dysfunction subtypes when compared to those with normal ventricular function. Bottom: Keeping ventricular function constant, neurological subgroups had significantly higher TAPSE/TRV ratios when compared to the cardiovascular subgroups. No significant difference in TAPSE/TRV ratios between cardiovascular, septic, and respiratory subgroups

In multivariate analysis, keeping diagnostic category constant, the TAPSE/TRV ratio was significantly lower in LV, RV and biventricular dysfunction subtypes when compared to those with normal ventricular function. The extent was not as large in isolated LV dysfunction, however **(**Fig. 4**).** While keeping ventricular function constant, neurological subgroups had significantly higher TAPSE/TRV ratios when compared to the cardiovascular subgroups. There was no significant difference in TAPSE/TRV ratios between cardiovascular, septic, and respiratory subgroups **(**Fig. 4**).**

### Prognostic relevance of TAPSE/TRV ratio in critically ill patients

**Fig. 5:**
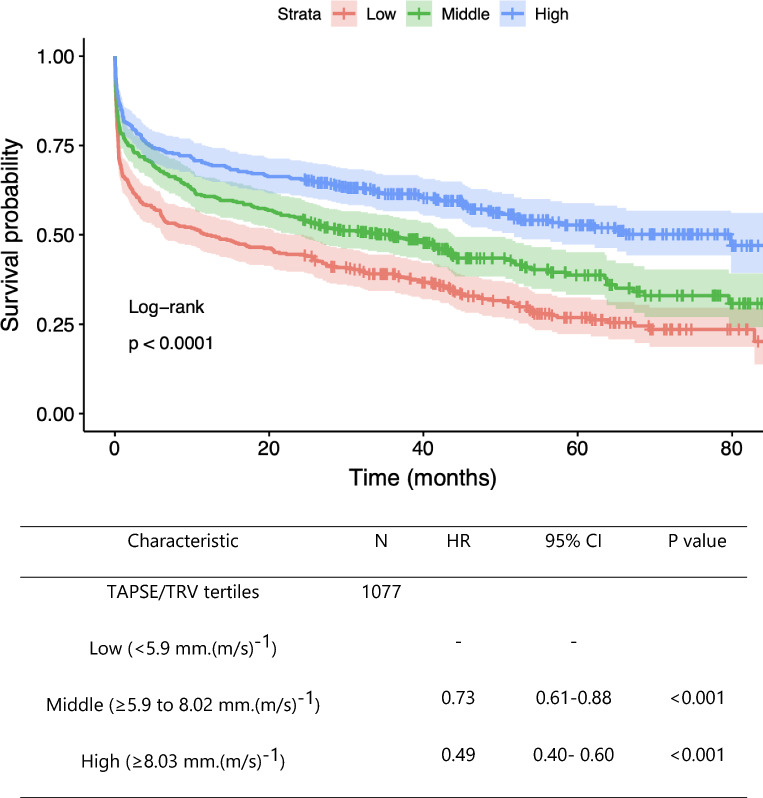
Kaplan–Meier survival analysis and Cox hazard regression model by TAPSE/TRV tertile in 1077 patients. Log rank < 0.0001. HR = hazard ratio; 95% CI = 95% confidence interval

There was an inverse relationship with TAPSE/TRV ratio and ICU survival. Higher TAPSE/TRV ratios were independently associated with lower risk of death in ICU after multivariate analysis (HR 0.927 [0.872–0.985], p < 0.05, *R*^2^ 0.155) **(**Additional file 1: Table S3). In a sequential multivariate analysis prediction model that included age, sex, invasive ventilation status, chronic cardiovascular illness, chronic respiratory illness, LV systolic function and APACHE 3 score as covariates only APACHE 3 score, chronic cardiovascular disease and TAPSE/TRV ratio remained independently associated with ICU mortality (Additional file 1: Table S3). The prognostic significance of TAPSE/TRV was confirmed by Kaplan–Meier analysis which showed lower survival in the low and middle TAPSE/TRV tertiles compared to high TAPSE/TRV tertile (log rank *p* < 0.0001) **(**Fig. 5**).**

**Fig. 6:**
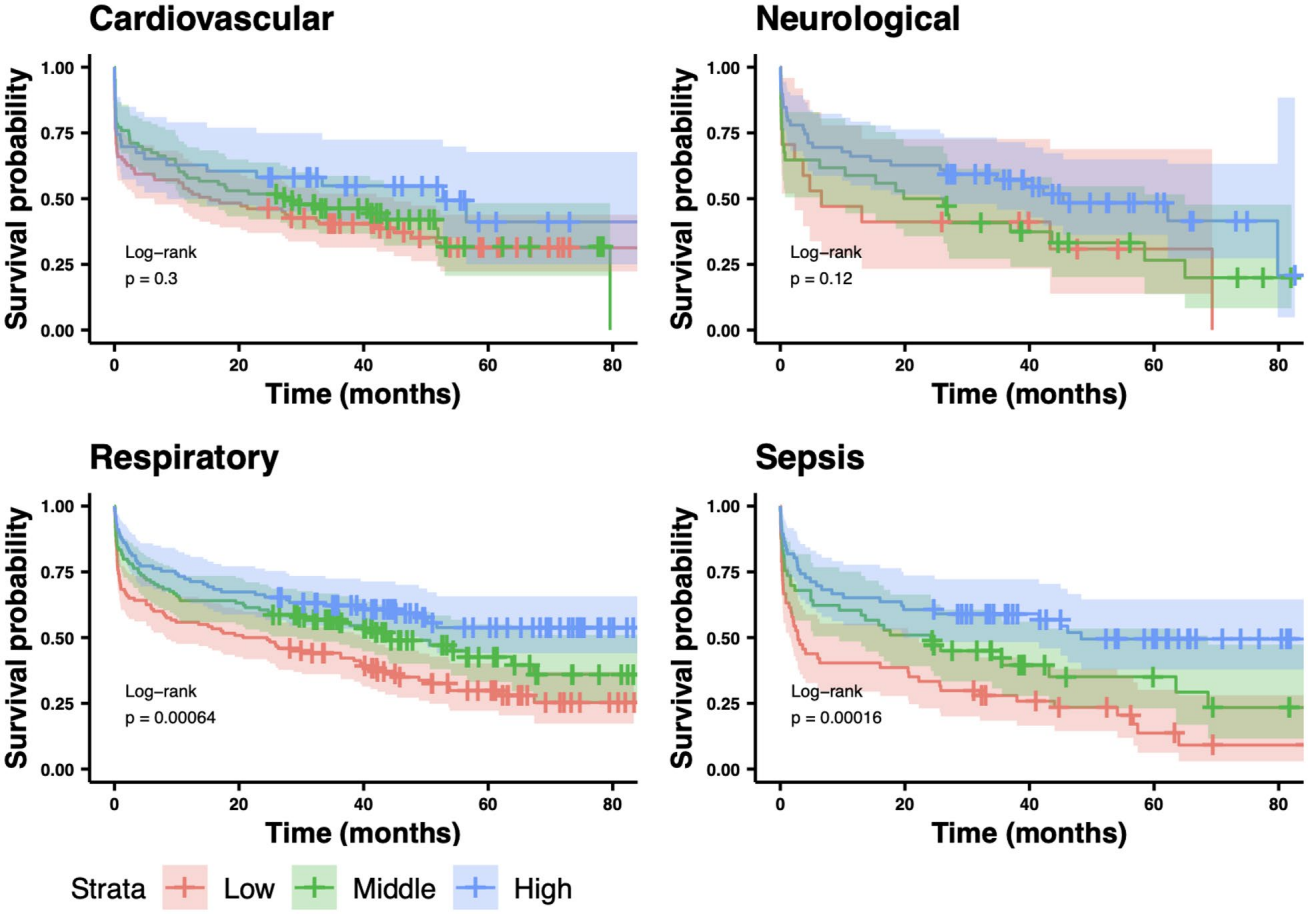
Kaplan–Meier survival curves with 95% confidence intervals as a function of the TAPSE/TRV tertile across different APACHE diagnostic subgroups. In respiratory and sepsis subgroups overall survival was lower in the low and middle TAPSE/TRV tertiles compared to high tertile groups (log rank *p* = 0.00064 and *p* = 0.000016, respectively)

**Fig. 7:**
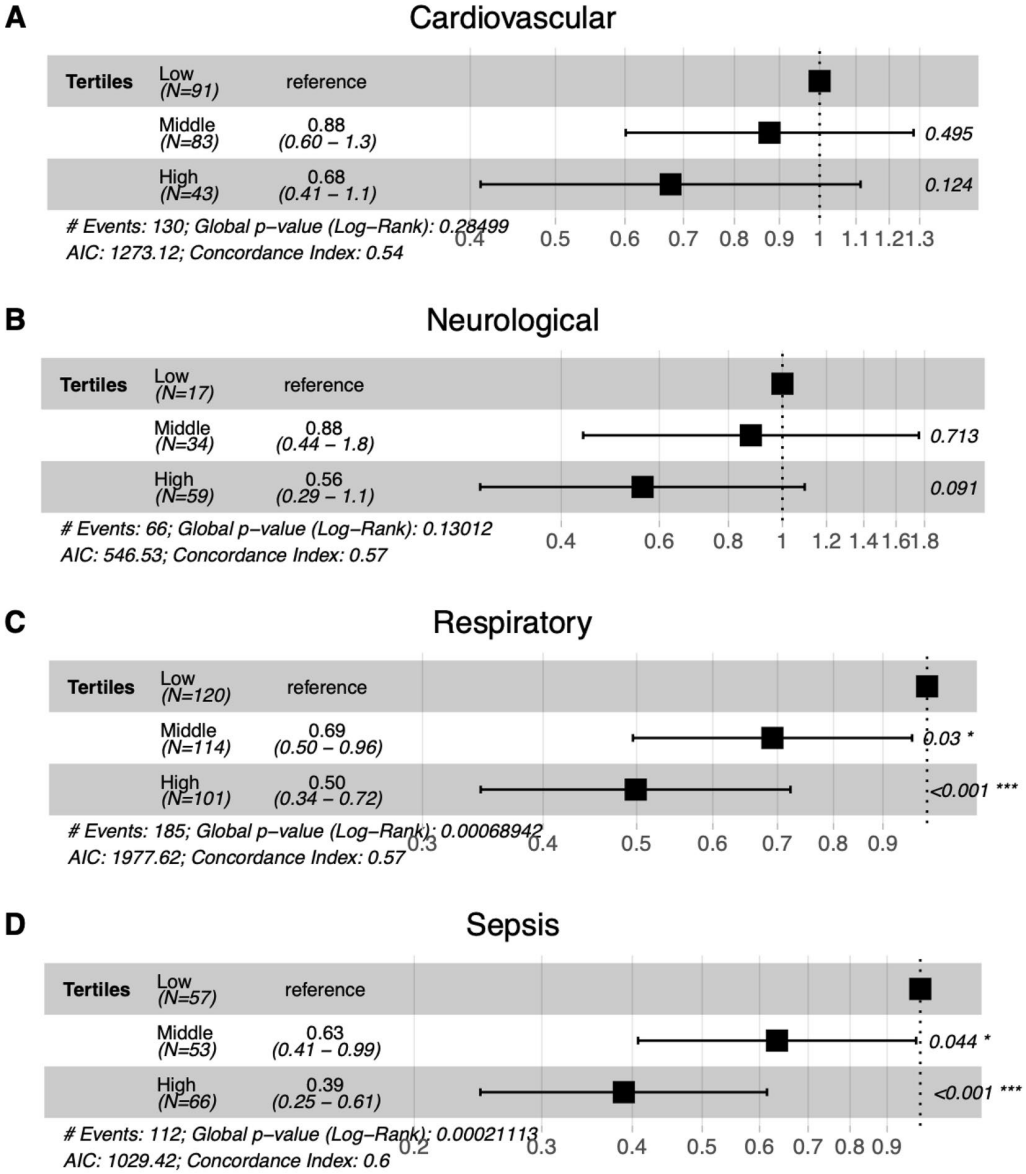
Cox proportional model hazard ratio for survival among TAPSE/TRV tertiles across diagnostic subgroups. The middle and high TAPSE/TRV tertiles in respiratory subgroups had a significantly lower risk of death compared to the lower tertile (HR = 0.69 [0.50, 0.96], *p* = 0.03 and HR = 0.50 [0.34,0.72], *p* < 0.001), respectively). The high and middle TAPSE/TRV tertiles in sepsis categories had significantly lower risk of death than those in the low tertile (HR = 0.63 [0.41,0.99], *p *0.044 and HR 0.39 [0.25, 0.61, *p* < 0.001], respectively)

Figure 6 shows the Kaplan–Meier curves for TAPSE/TRV tertiles across different diagnostic subgroups. The TAPSE/TRV ratio was not discriminative in neurological or cardiovascular subgroups, showing no difference in survival between TAPSE/TRV tertiles. By contrast, in the respiratory subgroups, the middle and high TAPSE/TRV tertile had a significantly lower risk of death compared to the lower tertile (HR = 0.69 [0.50, 0.96], *p* = 0.03 and HR = 0.50 [0.34,0.72], *p* < 0.001), respectively). In septic subgroups, the high and middle TAPSE/TRV tertile had significantly lower risk of death than those in the low tertile (HR = 0.63 [0.41, 0.99], *p* 0.044 and HR 0.39 [0.25, 0.61, *p* < 0.001], respectively. The corresponding Cox proportional hazard model results are shown in Fig. 7**.**

## Discussion

Our study reports a strong inverse relationship in ICU patients between TAPSE/TRV ratio and robust short- and long-term mortality data. Patients in the middle and low TAPSE/TRV ratio tertiles had worse long-term prognosis when compared to the high tertile. This indicates the potential for TAPSE/TRV ratio to be used in evaluation and risk prediction in ICU patients. TAPSE/TRV ratios below 5.9 mm.(m/s)^−1^, representing the most severe RV-PA uncoupling, had a significantly higher short- and long-term mortality than those with higher TAPSE/TRV ratios.

RV longitudinal function is impacted by LV systolic function and changes in LV ejection fraction correlate strongly with changes in TAPSE [25, 31]. In our analysis, those with isolated LV dysfunction had worse RV–PA coupling (lower TASPE/TRV ratios) than those with normal ventricular function, which is in keeping with previous studies [26, 32]. However, as expected, compared to those with biventricular dysfunction the impact of isolated LV dysfunction on RV-PA coupling was not as strong. In addition, our analysis showed that RV-PA uncoupling correlated with larger LA size and higher E/e’. These findings signal that raised left atrial pressure (LAP) could play a role in RV-PA uncoupling, which is in keeping with the recognized adverse influence of high LAP on pulmonary vascular remodeling and RV performance in heart failure cohorts [33, 34].

When adjusted for ventricular function the TAPSE/TRV ratio was not influenced by sepsis, cardiovascular or respiratory diagnoses, highlighting the potential robustness of this marker across a spectrum of critically unwell patients. Our analysis showed that TAPSE/TRV ratios may have a stronger risk stratification role in septic and respiratory diagnostic subgroups when compared to cardiovascular subgroups and neurological subgroups.

It was surprising that TAPSE/TRV ratios were not prognostic in the cardiovascular subgroup. This may be partially explained by a possibly sicker cohort in the cardiovascular high tertile group as evidenced by higher proportions of LV and biventricular dysfunction in this tertile (30%, compared to 16% and 14% in the sepsis and respiratory high tertile groups, respectively). The lack of RAP measurement may have influenced the proportion in high tertile with pseudo-normal TAPSE/TRV, where RAP inclusion may have reduced the coupling ratio. Further, in patients with RV failure (or biventricular failure), the range of TAPSE/TRV ratio could be wide depending on the relative magnitude of RV function (TAPSE) and the pressure generated by the failing RV (TRV). It could be that the use of TAPSE, limited by angle and loading conditions, as opposed to other less load-dependent surrogates of RV contractility such as RV free wall strain [35], performs less well in critically ill cardiovascular subgroups; however, this requires further investigation.

Rako et al. discuss the prognostic utility of several non-invasive RV-PA coupling parameters that show varying prognostic power depending on the clinical subgroup investigated [11], and it is likely that this performance variability extends to critically ill subgroups. To our knowledge, only TAPSE/PASP and RV fractional area change (FAC)/PASP have undergone validation against invasive gold standard Ees/Ea in heart failure [36] and pre-capillary PH cohorts [15]. Prospective studies evaluating non-invasive RV-PA coupling surrogates to invasive Ees/Ea could enable a better understanding of the therapeutic and prognostic utility of RV-PA coupling across pre-specified critically ill subgroups (e.g., acute heart failure, ARDS, sepsis).

Within respiratory subgroups, a significant proportion of patients (36%) had normal ventricular function, but low TAPSE/TRV ratios that was associated with higher mortality. This likely reflects the higher TRV and, thus, incidence of PH in this cohort. It suggests a particularly strong prognostic role of RV–PA coupling surrogates in patients admitted to ICU with primary respiratory illness. An uncoupled RV in association with large increases in TRV may be partly explained by its acute afterload sensitivity in an otherwise healthy RV [7, 37]. Importantly, almost half (48%) of the respiratory subgroup were ventilated and while ventilation did not appear to have a significant impact on TAPSE/TRV ratio, the applied stratification of the TAPSE/TRV ratio to all ventilated and non-ventilated patients is a limitation to generalize the results, or draw firm conclusion given detailed respiratory mechanics data are unknown.

There were significant proportions of pseudo-normal TAPSE/TRV ratio with high TRV (24%, 21%, and 19%, in respiratory, cardiovascular, and sepsis subgroups, respectively). There are some possible causes for this, (1) lack of RAP measurement, (2) the TRV may only be mildly increased, and not high enough to reduce the ratio, (3) although TRV has increased, the RV may be compensating by increasing contraction (higher TAPSE) and this may represent RV compensation or early RV maladaptation but with preserved coupling [10, 11]. If the latter is significant, then it could help partly explain the improved survival in those in higher TAPSE/TRV tertiles, including pseudo-normal groups.

Lower TAPSE/TRV ratios were found in those with greater degrees of right heart dilatation and TR, which is in keeping with findings in PH cohorts [14]. Lower TAPSE/PASP coupling ratios were independently associated with decreased survival in patients with TR undergoing transcatheter repair, where it outperformed TAPSE or PASP alone [38]. However, given the limitations of both TAPSE and PASP measurement in severe TR [11, 39, 40], other coupling surrogates (e.g., stroke volume/end systolic volume) using 3D RV volumes have been investigated and show prognostic utility [41].

In our analysis, TAPSE/TRV ratios were taken from a single snapshot in time and the value of serial assessment of TAPSE/TRV in critically ill patients remains unknown. In patients with pre-capillary PH, dobutamine stress testing has been shown to worsen RV-PA coupling (assessed using TAPSE/PASP) indicating exhausted contractile reserve [42]. Given dobutamine is often a first-choice inodilator in the context of RV dysfunction, serial assessment of RV–PA coupling could help earlier identification of subgroups with exhausted compensatory mechanisms, thus directing treatments toward afterload reduction and possibly risk stratifying those who may benefit from mechanical RV support.

It is unknown whether interventions to improve RV–PA coupling improves patient outcomes and further prospective investigation is needed. However, the robust association between impaired RV–PA coupling (lower TAPSE/TRV ratio) and mortality in this study suggests therapies aimed at improving RV–PA coupling may improve outcomes. Inotropic agents such as milrinone and levosimendan (among others) can enhance RV–PA coupling allowing time to resolve the underlying disease process [43, 44]. Other therapeutic interventions might include pulmonary vasodilators to reduce pulmonary pressures, or treatments to minimize RV dilatation and adverse interventricular dependence by reducing filling pressures and RV afterload using diuretics, prone ventilation, and mechanical supports [32, 45, 46].

## Limitations

Limitations in this study are inherent to its retrospective, single-center design. Several factors following ICU discharge are likely to impact mortality outcomes that are unaccounted for in this analysis. However, this is a large dataset with robust mortality end points.

Only those who had a TTE were included in the study introducing possible selection bias; however, TTE data were obtained for clinical indications, which increases the applicability of this data to routine practice in an ICU environment. Studies were not independently reviewed, and measurements were not repeated, with original measurements assumed to be correct. TRV was not measurable in a significant proportion of patients (39.7%), similar rates to a study in only mechanically ventilated patients [47], and we cannot exclude the possibility that inclusion of these patients could have influenced the results. Similarly, we did not confirm the presence of PH with right heart catheterization in all patients, although the robust findings of these results show the utility of this simplified marker even in the absence of right heart catheter confirmation of PA pressure. A main strength of the TAPSE/TRV ratio is its simplicity for use at the bedside because it does not rely on estimation of RAP, which is a known limitation of echocardiography in the critically ill [23, 48]. However, it is also a significant limitation that RAP is not included, negating the impact of preload status on right heart performance [5, 49]. This may have impacted accuracy of findings, particularly in relation to patients with pseudo-normal TAPSE/TRV ratios. Furthermore, only TAPSE/TRV was explored as a surrogate RV-PA coupling metric despite many other non-invasive coupling ratios described [11, 50]. TAPSE is angle dependent and affected by the longitudinal rotation of the heart, particularly in pressure overloaded states, where it can overestimate systolic function [51]. Furthermore, TAPSE measures a single dimension of RV function, negating the bellows like contraction of the RV free wall and peristaltic contraction of the RV outflow to overall RV systolic function [37].

Important chronic comorbidities of patients were unknown including pre-existing valvular disease, PH, ventricular systolic and diastolic dysfunction. The APACHE diagnostic subgroups are broad and resulting heterogeneity within subgroups is unaccounted for. Importantly, the amount of hemodynamic and ventilatory support is not reported including data on respiratory mechanics. The lack of a TTE on discharge also limits generalizability of the study findings. Semi-quantitative and quantitative echocardiographic parameters such as Simpson’s ejection fraction, ventricular and atrial chamber size, cardiac output, RV end diastolic/LV end diastolic area ratio, RV eccentricity index, acute cor pulmonale, RV free wall thickness, RV fractional area change and RV diastolic data were not available in sufficient numbers. Further, the evaluation of LV dysfunction by visual assessment in a single center introduces bias, though it was performed by experienced clinicians with advanced echo qualification and represents everyday practices.

## Summary

We have demonstrated that TAPSE/TRV ratio is a useful marker that can be applied reliably and simply to routine care of critically ill patients in an ICU environment, showing strong independent association with short and long-term mortality. This study adds to the growing body of literature that normalizing RV systolic function for afterload (assessing RV–PA coupling) can aid risk stratification in critically ill patients. Prospective evaluation of routine application of TAPSE/TRV ratio to help guide ICU management decisions is needed.

### Supplementary Information

**Additional file 1.** **Supplemental figure 1.** Concept of TAPSE/TRV ratio. **Supplemental figure 2.** TAPSE/TRV tertiles. **Supplemental Table 1.** Echocardiographic parameters across TAPSE/TRV tertiles. **Supplemental figure 3.** APACHE diagnostic categories receiving a Transthoracic Echocardiogram. **Supplemental figure 4.** Relationship of TAPSE/TRV to right heart chamber dilatation and valvular regurgitation severity. **Supplemental Figure 5.** TAPSE/TRV tertiles across ventricular function subgroups stratified by diagnostic category. **Supplemental Table 2.** Patient characteristics across four diagnostic subgroups. **Supplemental Figure 6.** Kaplan-Meier survival curves and Cox hazard regression across diagnostic subgroups. **Supplemental figure 7.** Number of patients in each ‘TAPSE/TRV ratio - TAPSE - TRV’ category across diagnostic groups. **Supplemental Table 3.** Sequential Cox hazard multivariate analysis.

## Data Availability

The datasets used and/or analyzed during the current study are available from the corresponding author on reasonable request.

## Abbreviations

RV-PA: Coupling right ventricular-pulmonary arterial coupling
TAPSE: Tricuspid annular plane systolic excursion
TRV: Tricuspid regurgitant maximal velocity
RV: Right ventricle
RA: Right atrium
PA: Pulmonary artery
LV: Left ventricle
LA: Left atrium
Echo: Echocardiography
LVEDD: Left ventricular end diastolic diameter
LVESD: Left ventricular end systolic diameter
LVEF: Left ventricular ejection fraction
E/e’: Early mitral inflow velocity inflow by pulsed wave Doppler/average mitral annular velocity by tissue Doppler imaging
E/A: Early mitral inflow velocity by pulsed wave Doppler/ mitral inflow velocity from atrial contraction
PWD: Pulsed wave Doppler
FS: Fractional shortening
LVEF: Left ventricular ejection fraction
MR: Mitral regurgitation
AR: Aortic regurgitation
TR: Tricuspid regurgitation
FAC: Fractional area change

## References

[1] Lanspa MJ, Cirulis MM, Wiley BM, Olsen TD, Wilson EL, Beesley SJ (2021). Right ventricular dysfunction in early sepsis and septic shock. Chest.

[2] Mekontso Dessap A, Boissier F, Charron C, Bégot E, Repessé X, Legras A (2016). Acute cor pulmonale during protective ventilation for acute respiratory distress syndrome: prevalence, predictors, and clinical impact. Intensive Care Med.

[3] Vieillard-Baron A, Naeije R, Haddad F, Bogaard HJ, Bull TM, Fletcher N (2018). Diagnostic workup, etiologies and management of acute right ventricle failure: a state-of-the-art paper. Intensive Care Med.

[4] Noordegraaf AV, Chin KM, Haddad F, Hassoun PM, Hemnes AR, Hopkins SR (2019). Pathophysiology of the right ventricle and of the pulmonary circulation in pulmonary hypertension: an update. Eur Respir J.

[5] Pinsky MR (2016). The right ventricle: interaction with the pulmonary circulation. Crit Care.

[6] Houston BA, Brittain EL, Tedford RJ (2023). Right ventricular failure. N Engl J Med.

[7] Vonk Noordegraaf A, Westerhof BE, Westerhof N (2017). The Relationship between the right ventricle and its load in pulmonary hypertension. J Am Coll Cardiol.

[8] Sunagawa K, Maughan WL, Burkhoff D, Sagawa K (1983). Left ventricular interaction with arterial load studied in isolated canine ventricle. Am J Physiol Heart Circ Physiol.

[9] Bernardo RJ, Haddad F, Couture EJ, Hansmann G, de Jesus Perez VA, Denault AY (2020). Mechanics of right ventricular dysfunction in pulmonary arterial hypertension and heart failure with preserved ejection fraction. Cardiovasc Diagn Ther.

[10] Tello K, Dalmer A, Axmann J, Vanderpool R, Ghofrani HA, Naeije R (2019). Reserve of right ventricular-arterial coupling in the setting of chronic overload. Circ Heart Fail.

[11] Rako ZA, Kremer N, Yogeswaran A, Richter MJ, Tello K (2023). Adaptive versus maladaptive right ventricular remodelling. ESC Heart Fail.

[12] Hsu S (2019). Coupling right ventricular-pulmonary arterial research to the pulmonary hypertension patient bedside. Circ Heart Fail.

[13] Zochios V, Yusuff H, Schmidt M (2023). Acute right ventricular injury phenotyping in ARDS. Intensive Care Med.

[14] Tello K, Axmann J, Ghofrani HA, Naeije R, Narcin N, Rieth A (2018). Relevance of the TAPSE/PASP ratio in pulmonary arterial hypertension. Int J Cardiol.

[15] Tello K, Wan J, Dalmer A, Vanderpool R, Ghofrani HA, Naeije R (2019). Validation of the tricuspid annular plane systolic excursion/systolic pulmonary artery pressure ratio for the assessment of right ventricular-arterial coupling in severe pulmonary hypertension. Circ Cardiovasc Imaging.

[16] Guazzi M (2018). Use of TAPSE/PASP ratio in pulmonary arterial hypertension: an easy shortcut in a congested road. Int J Cardiol.

[17] Guazzi M, Dixon D, Labate V, Beussink-Nelson L, Bandera F, Cuttica MJ (2017). RV contractile function and its coupling to pulmonary circulation in heart failure with preserved ejection fraction: stratification of clinical phenotypes and outcomes. JACC Cardiovasc Imaging.

[18] Richter MJ, Peters D, Ghofrani HA, Naeije R, Roller F, Sommer N (2020). Evaluation and prognostic relevance of right ventricular-arterial coupling in pulmonary hypertension. Am J Respir Crit Care Med.

[19] Anastasiou V, Papazoglou AS, Moysidis DV, Daios S, Barmpagiannos K, Gossios T (2023). The prognostic impact of right ventricular-pulmonary arterial coupling in heart failure: a systematic review and meta-analysis. Heart Fail Rev.

[20] D’Alto M, Marra AM, Severino S, Salzano A, Romeo E, De Rosa R (2020). Right ventricular-arterial uncoupling independently predicts survival in COVID-19 ARDS. Crit Care.

[21] Zhang H, Lian H, Zhang Q, Chen X, Wang X, Liu D (2020). Prognostic implications of tricuspid annular plane systolic excursion/pulmonary arterial systolic pressure ratio in septic shock patients. Cardiovasc Ultrasound.

[22] Lyhne MD, Kabrhel C, Giordano N, Andersen A, Nielsen-Kudsk JE, Zheng H (2021). The echocardiographic ratio tricuspid annular plane systolic excursion/pulmonary arterial systolic pressure predicts short-term adverse outcomes in acute pulmonary embolism. Eur Heart J Cardiovasc Imaging.

[23] Alavi-Moghaddam M, Kabir A, Shojaee M, Manouchehrifar MMM (2017). Ultrasonography of inferior vena cava to determine central venous pressure: a meta-analysis and meta-regression. Acta Radiol.

[24] Vicenzi M, Caravita S, Rota I, Casella R, Deboeck G, Beretta L (2022). The added value of right ventricular function normalized for afterload to improve risk stratification of patients with pulmonary arterial hypertension. PLoS ONE.

[25] Lamia B, Teboul JL, Monnet X, Richard C, Chemla D (2007). Relationship between the tricuspid annular plane systolic excursion and right and left ventricular function in critically ill patients. Intensive Care Med.

[26] Guazzi M, Bandera F, Pelissero G, Castelvecchio S, Menicanti L, Ghio S (2013). Tricuspid annular plane systolic excursion and pulmonary arterial systolic pressure relationship in heart failure: an index of right ventricular contractile function and prognosis. Am J Physiol Heart Circ Physiol.

[27] Strange G, Celermajer DS, Marwick T, Prior D, Ilton M, Codde J (2018). The National Echocardiography Database Australia (NEDA): rationale and methodology. Am Heart J.

[28] Zoghbi WA, Adams D, Bonow RO, Enriquez-sarano M, Thomas JD (2017). Recommendations for noninvasive evaluation of native valvular regurgitation. J Am Soc Echocardiogr.

[29] Benchimol EI, Smeeth L, Guttmann A, Harron K, Moher D, Peteresen I (2015). The REporting of studies Conducted using Observational Routinely-collected health Data (RECORD) statement. PLoS Med.

[30] von Elm E, Altman DG, Egger M, Pocock SJ, Gøtzsche PC, Vandenbroucke JP (2014). The strengthening the reporting of observational studies in epidemiology (STROBE) statement: guidelines for reporting observational studies. Int J Surg.

[31] López-Candales A, Rajagopalan N, Saxena N, Gulyasy B, Edelman K, Bazaz R (2006). Right ventricular systolic function is not the sole determinant of tricuspid annular motion. Am J Cardiol.

[32] Jentzer JC, Anavekar NS, Reddy YNV, Murphree DH, Wiley BM, Oh JK (2021). Right ventricular pulmonary artery coupling and mortality in cardiac intensive care unit patients. J Am Heart Assoc.

[33] Guazzi M, Ghio S, Adir Y (2020). Pulmonary Hypertension in HFpEF and HFrEF: JACC Review Topic of the Week. J Am Coll Cardiol.

[34] Humbert M, Kovacs G, Hoeper MM, Badagliacca R, Berger RMF, Brida M (2022). 2022 ESC/ ERS guidelines for the diagnosis and treatment of pulmonary hypertension developed by the task force for the diagnosis and treatment of (ESC) and the European Respiratory Society (ERS). Eur Heart J.

[35] Lang RM, Badano LP, Victor MA, Afilalo J, Armstrong A, Ernande L (2015). Recommendations for cardiac chamber quantification by echocardiography in adults: an update from the American Society of Echocardiography and the European Association of Cardiovascular Imaging. J Am Soc Echocardiogr.

[36] Schmeisser A, Rauwolf T, Groscheck T, Kropf S, Luani B, Tanev I (2021). Pressure-volume loop validation of TAPSE/PASP for right ventricular arterial coupling in heart failure with pulmonary hypertension. Eur Heart J Cardiovasc Imaging.

[37] Haddad F, Hunt SA, Rosenthal DN, Murphy DJ (2008). Right ventricular function in cardiovascular disease, part I: Anatomy, physiology, aging, and functional assessment of the right ventricle. Circulation.

[38] Brener MI, Lurz P, Hausleiter J, Rodés-Cabau J, Fam N, Kodali SK (2022). Right ventricular-pulmonary arterial coupling and afterload reserve in patients undergoing transcatheter tricuspid valve repair. J Am Coll Cardiol.

[39] Howard LS, Grapsa J, Dawson D, Bellamy M, Chambers JB, Masani ND (2012). Echocardiographic assessment of pulmonary hypertension: standard operating procedure. Eur Respir Rev.

[40] Ancona F, Melillo F, Calvo F, Attalla El Halabieh N, Stella S, Capogrosso C (2021). Right ventricular systolic function in severe tricuspid regurgitation: Prognostic relevance of longitudinal strain. Eur Heart J Cardiovasc Imaging.

[41] Gavazzoni M, Badano LP, Cascella A, Heilbron F, Tomaselli M, Caravita S (2023). Clinical value of a novel three-dimensional echocardiography-derived index of right ventricle-pulmonary artery coupling in tricuspid regurgitation. J Am Soc of Echocardiogr.

[42] Ghio S, Fortuni F, Greco A, Turco A, Lombardi C, Scelsi L, et al. Dobutamine stress echocardiography in pulmonary arterial hypertension. Int J Cardiol [Internet]. 2018;270:331–5. Available from: 10.1016/j.ijcard.2018.06.032 10.1016/j.ijcard.2018.06.032 29903514

[43] Price LC, Wort SJ, Finney SJ, Marino PS, Brett SJ (2010). Pulmonary vascular and right ventricular dysfunction in adult critical care: current and emerging options for management: a systematic literature review. Crit Care.

[44] Lyhne MD, Dragsbaek SJ, Hansen JV, Schultz JG, Andersen A, Nielsen-Kudsk JE (2021). Levosimendan, milrinone, and dobutamine in experimental acute pulmonary embolism. Pulm Circ.

[45] Vieillard-Baron A, Charron C, Caille V, Belliard G, Page B, Jardin F (2007). Prone positioning unloads the right ventricle in severe ARDS. Chest.

[46] Petit M, Jullien E, Vieillard-Baron A (2022). Right ventricular function in acute respiratory distress syndrome: impact on outcome, respiratory strategy and use of veno-venous extracorporeal membrane oxygenation. Front Physiol.

[47] Mercado P, Maizel J, Beyls C, Kontar L, Orde S, Huang S (2019). Reassessment of the accuracy of cardiac doppler pulmonary artery pressure measurements in ventilated ICU patients: a simultaneous doppler-catheterization study. Crit Care Med.

[48] Jue J, Chung W, Schiller NB (1992). Does inferior vena cava size predict right atrial pressures in patients receiving mechanical ventilation?. J Am Soc Echocardiogr.

[49] Vieillard-Baron A, Pinsky MR (2021). The difficulty in defining right ventricular failure at the bedside and its clinical significance. Ann Intensive Care.

[50] Shahim B, Hahn RT (2021). Right ventricular-pulmonary arterial coupling and outcomes in heart failure and valvular heart disease. Structural Heart.

[51] Collier P, Xu B, Kusunose K, Phelan D, Grant A, Thavendiranathan P (2018). Impact of abnormal longitudinal rotation on the assessment of right ventricular systolic function in patients with severe pulmonary hypertension. J Thorac Dis.

