# Supplementary material for: The value of right ventricular to pulmonary arterial coupling in the critically ill: a National Echocardiography Database of Australia (NEDA) substudy
Source: Ann Intensive Care. 2024 Jan 16;14:10. doi: 10.1186/s13613-024-01242-0 (PMC10792157; doi:10.1186/s13613-024-01242-0)
Supplement: Supplementary file 1 — Additional file 1. Supplemental figure 1. Concept of TAPSE/TRV ratio. Supplemental figure 2. TAPSE/TRV tertiles. Supplemental Table 1. Echocardiographic parameters across TAPSE/TRV tertiles. Supplemental figure 3. APACHE diagnostic categories receiving a Transthoracic Echocardiogram. Supplemental figure 4. Relationship of TAPSE/TRV to right heart chamber dilatation and valvular regurgitation severity. Supplemental Figure 5. TAPSE/TRV tertiles across ventricular function subgroups stratified by diagnostic category. Supplemental Table 2. Patient characteristics across four diagnostic subgroups. Supplemental Figure 6. Kaplan-Meier survival curves and Cox hazard regression across diagnostic subgroups. Supplemental figure 7. Number of patients in each ‘TAPSE/TRV ratio - TAPSE - TRV’ category across diagnostic groups. Supplemental Table 3. Sequential Cox hazard multivariate analysis. [file 13613_2024_1242_MOESM1_ESM.docx]

Supplementary material

**Supplemental figure 1. Concept of TAPSE/TRV ratio.**


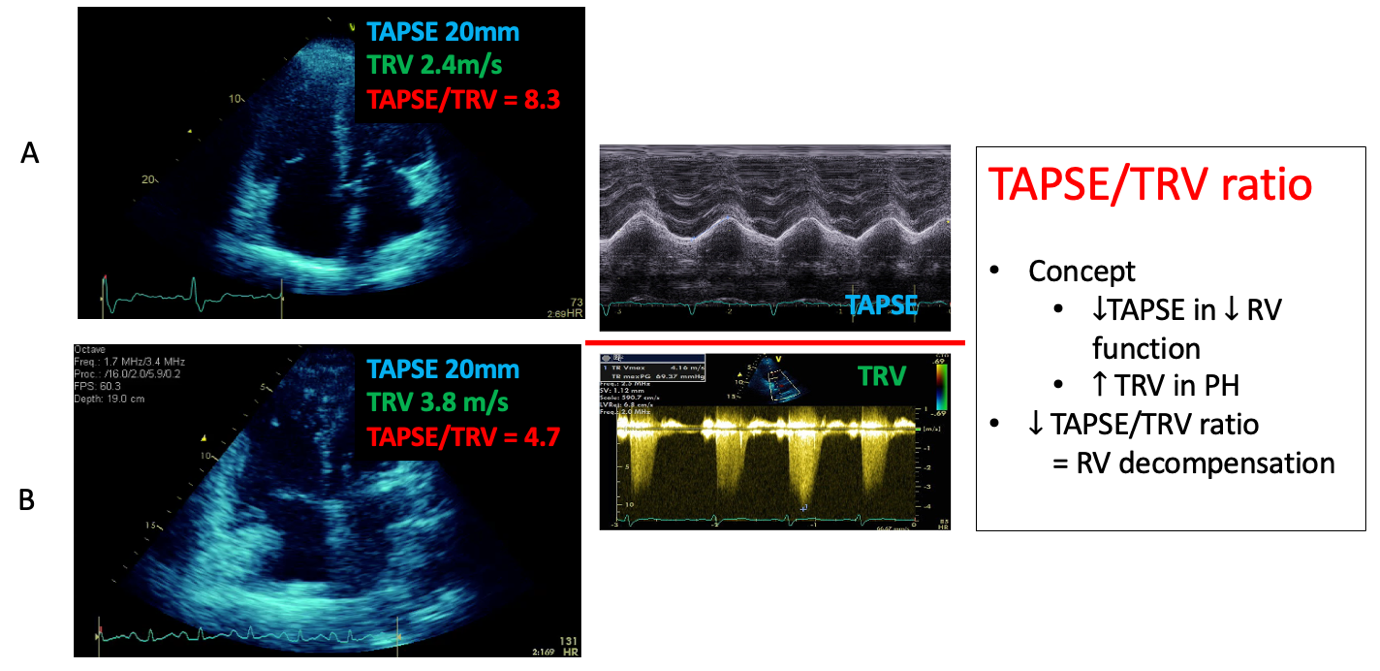


*Supplemental figure 1. Concept of TAPSE/TRV ratio. Time point A: Apical 4 chamber showing RV dilatation, TAPSE 20mm and TRV 2.4m/s with a TAPSE/TRV ratio of 8.3* mm.(m/s)^-1^ *indicating preserved RV-PA coupling. Time point B: Apical 4 chamber showing RV dilation with TAPSE 20mm and a higher TRV of 3.8m/s resulting in a lower TAPSE/TRV ratio of 4.7* mm.(m/s)^-1^*. From point A to B, TAPSE has not increased in response to increased RV afterload, signalling deteriorating RV-PA coupling with higher risk of further RV decompensation and RV failure.*

**Supplemental figure 2. TAPSE/TRV tertiles**


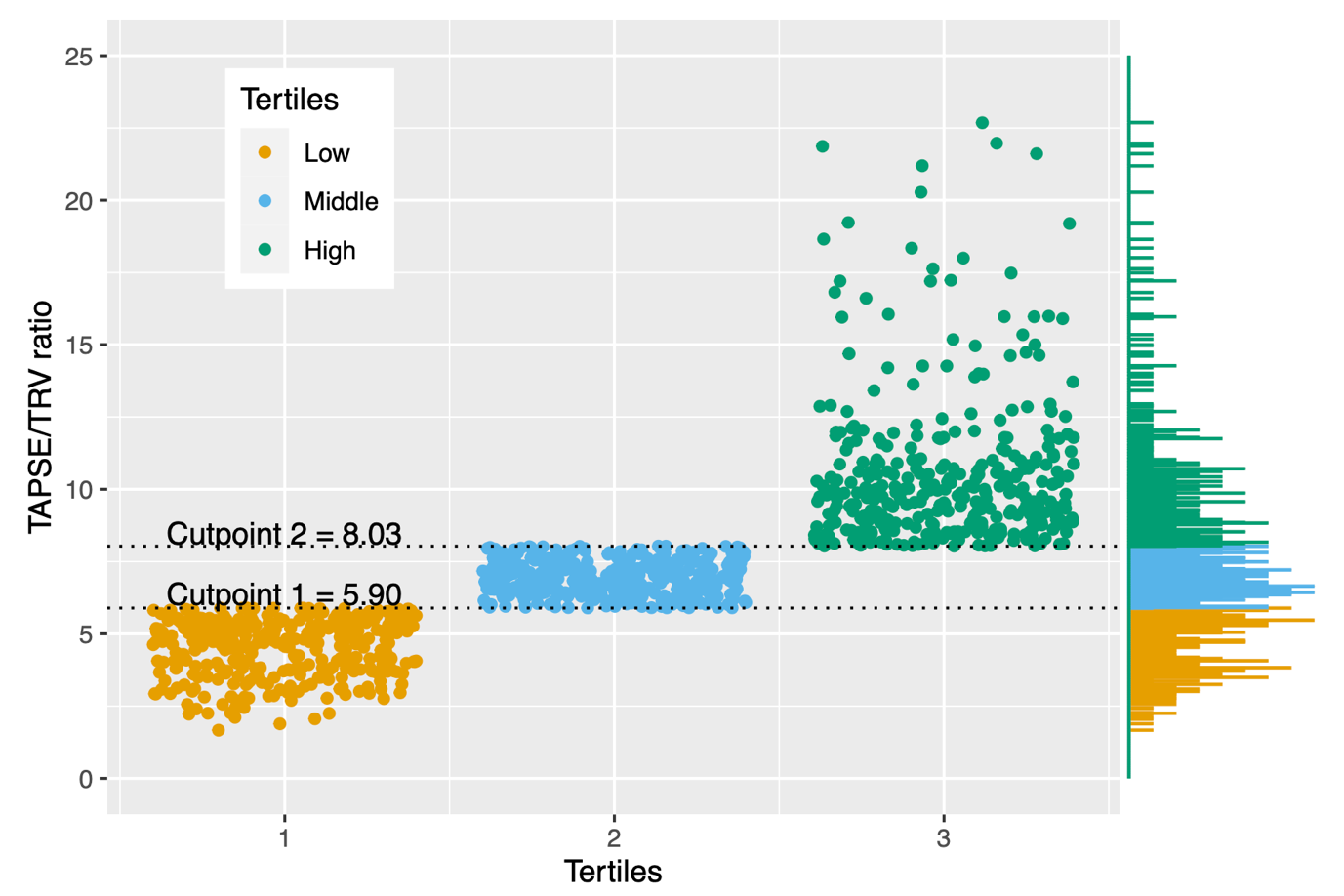


*Supplemental figure 2. TAPSE/TRV tertile group: low (<5.9 mm.(m/s)-1 ), middle (≥5.9 to 8.02 mm.(m/s)-1) and high (≥8.03 mm.(m/s)-1). Higher TAPSE/TRV ratios reflect better RV-PA coupling.*

**Supplemental Table 1. Echo parameters according to TAPSE/TRV tertile**

| **TAPSE/TRV Tertile** | | | |
| --- | --- | --- | --- |
| **Echo Parameter** | **Low (n=359)** | **Middle (n=359)** | **High (n=359)** |
| **LVEDD (cm) (n=944)** | 4.8 (4.2,5.5) | 4.7 (4.1,5.2) | 4.7 (4.2,5.2) |
| **LVESD (cm) (n=687)** | 3.5 (2.7,4.4) | 3.2 (2.7,3.8) | 3.1 (2.6, 3.6) |
| **FS (%) (n=687)** | 42 (36, 47) | 41 (35, 46) | 41 (37, 46) |
| **LVEF (Simpson’s) (n=384)** | 35 (25, 50) | 48 (35, 60) | 55 (45, 65) |
| **E/A ratio (n=756)** | 1.06 (0.76,1.6) | 1.09 (0.81, 1.42) | 1.07 (0.86, 1.35) |
| **E/e’ ratio (n=902)** | 10.0 (9.1,13.5) | 10.0 (8.4, 12.0) | 9.2 (7.1, 10.4) |
| **LA diameter (cm) (n=952)** | 4.0 (3.5, 4.6) | 3.7 (3.2, 4.1) | 3.6 (3.2, 3.9) |
| **TAPSE (mm)** | 13.7 (11.2, 16.6) | 19.7 (17.4, 21.7) | 24.0 (21.6, 27.3) |
| **TRV (m/s)** | 3.04 (2.64, 3.43) | 2.83 (2.50, 3.16) | 2.43 (2.10, 2.74) |
| **TAPSE/TRV (mm.(m/s)^-1^)** | 4.73 (3.81, 5.38) | 6.92 (6.44, 7.46) | 9.78 (8.83, 11.04) |
| **TR Severity (n=556)** |  |  |  |
| Trace/Mild | 117 (49%) | 141 (74%) | 114 (88%) |
| Mod/Severe | 120 (51%) | 46 (26%) | 15 (12%) |
| **MR Severity (n=419)** |  |  |  |
| Trace/Mild | 132 (74%) | 108 (84%) | 103 (92%) |
| Mod/Severe | 47 (26%) | 20 (16%) | 9 (8%) |
| **AR Severity (n=170)** |  |  |  |
| Trace/Mild | 62 (76%) | 36 (78%) | 34 (81%) |
| Mod/Severe | 20 (24%) | 10 (22%) | 8 (19%) |

*Supplemental Table 1. Echo parameters according to TAPSE/TRV tertile. LVEDD=Left ventricular end diastolic diameter; LVESD=Left ventricular end systolic diameter; FS= fractional shortening LVEF=Left ventricular ejection fraction; LA=left atrium; TRV =Tricuspid regurgitation maximal velocity; MR=Mitral regurgitation; AR= Aortic regurgitation. Median (IQR); n (%)*

**Supplemental figure 3. APACHE diagnostic categories receiving a Transthoracic Echocardiogram**

**
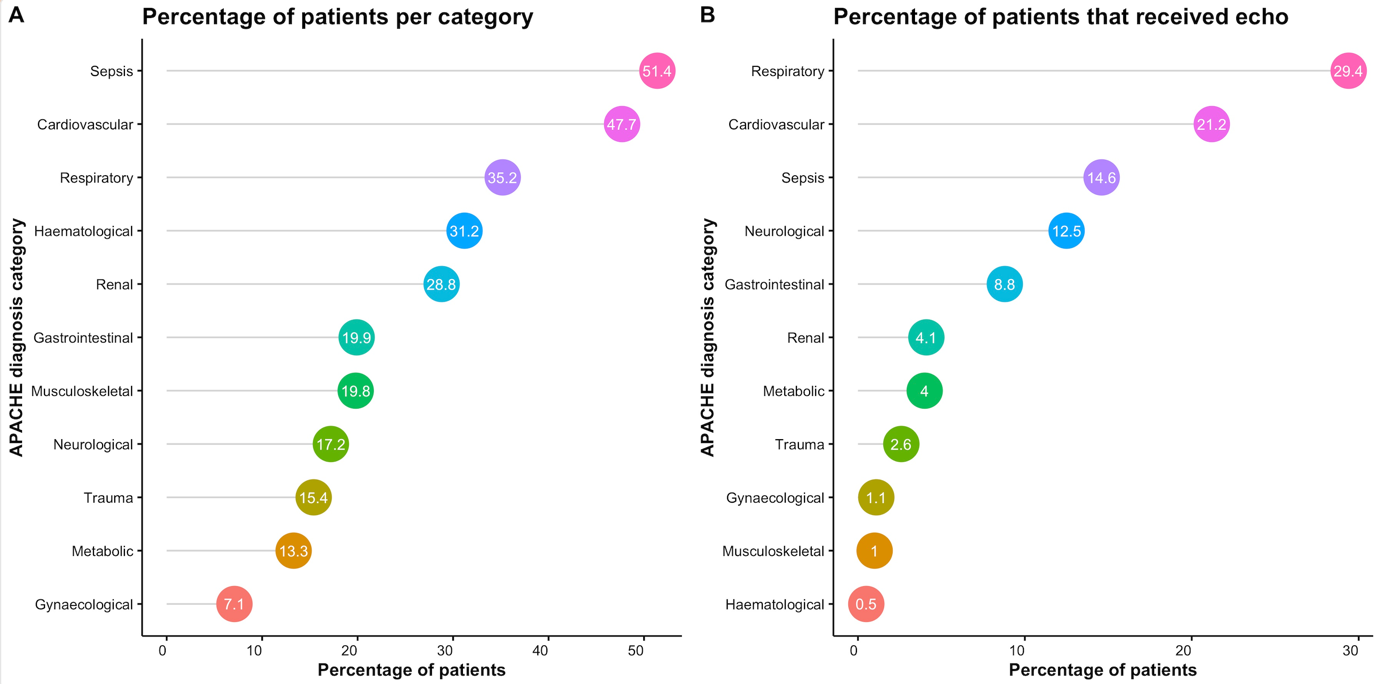
**

*Supplemental figure 3. Diagnostic categories in 1838 patients who had an indication for TTE are shown (A), alongside percentage who received a transthoracic echocardiogram (TTE) across diagnostic categories (B). TTE= transthoracic echocardiogram*

**Supplemental figure 4. Relationship of TAPSE/TRV to right heart chamber dilatation and valvular regurgitation severity**


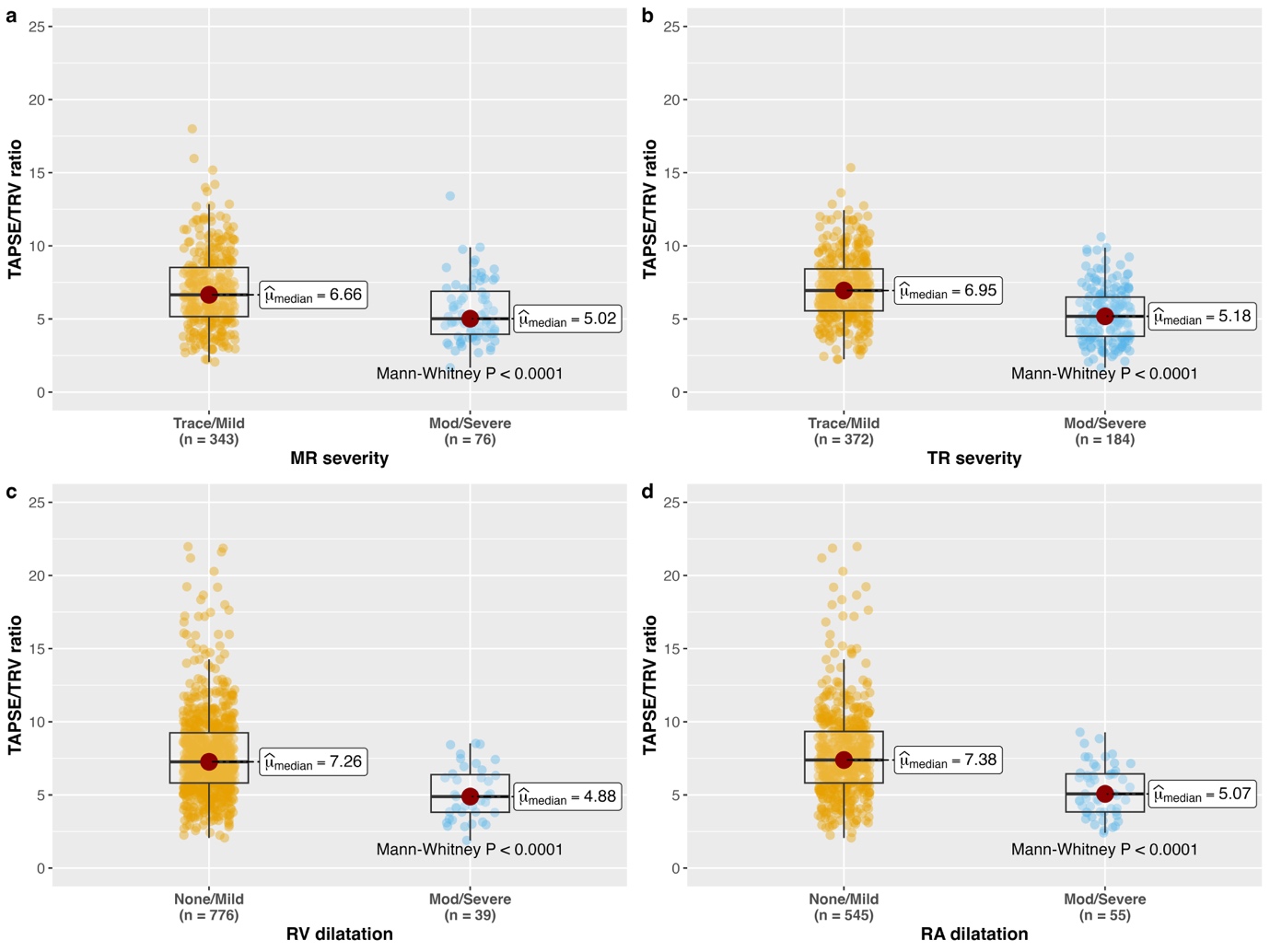


*Supplemental figure 4. Relationship of TAPSE/TRV with other echocardiographic parameters. TAPSE/TRV stratified by (a) MR severity (n=419) (b) TR severity (n=556) (c) RV dilatation (n=815) (d) RA dilatation (n=600)*

**Supplemental Figure 5. TAPSE/TRV tertiles across ventricular function subgroups stratified by diagnostic category**


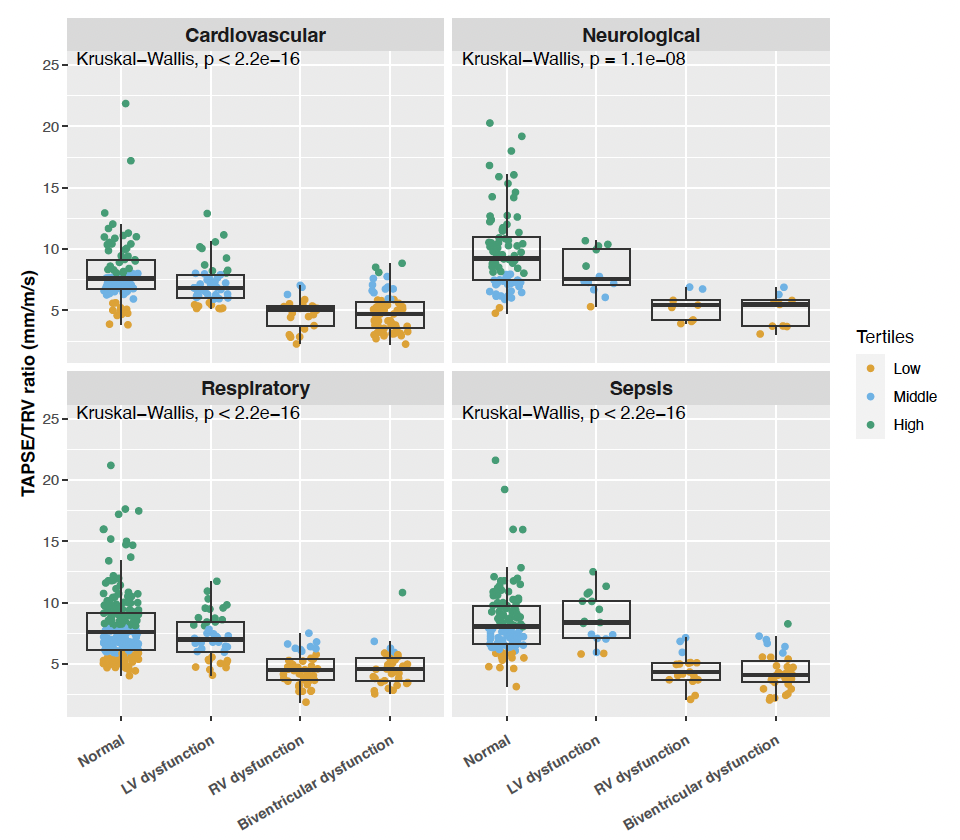


*Supplemental Figure 5. Relationship between TAPSE/TRV tertiles across ventricular function subgroups stratfied by diagnostic category. Across all diagnostic subgroups, lower TAPSE/TRV ratios are found in RV and biventricular dysfunction as compared to normal or isolated LV dysfunction.*

**Supplemental Table 2. Patient characteristics across four diagnostic subgroups**

**
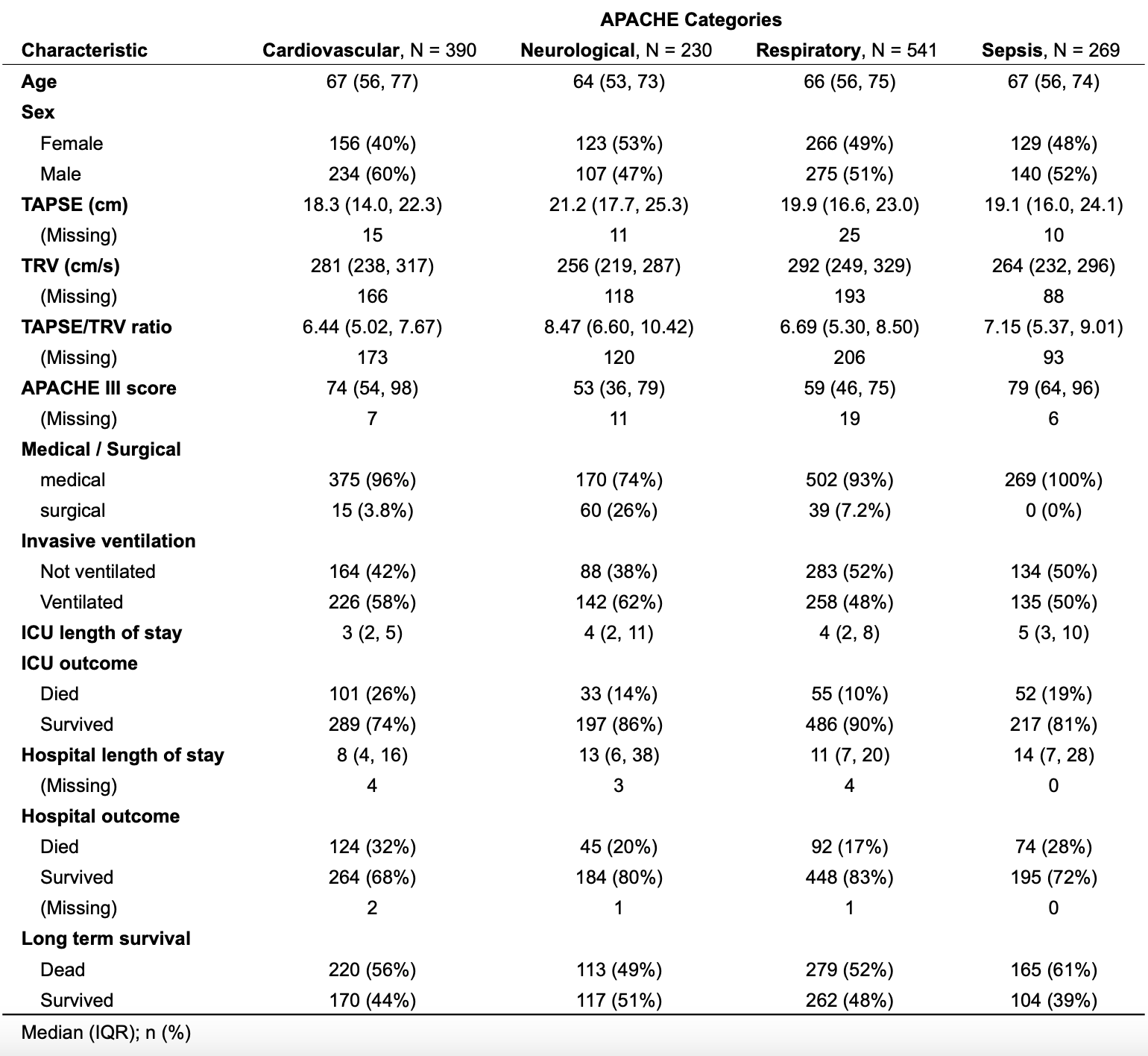
**

*Supplemental Table 2. Patient characteristics and TAPSE/TRV ratios across APACHE diagnostic subgroups. Median (IQR).*

**Supplemental Figure 6. Kaplan-Meier survival curves and Cox hazard regression across diagnostic subgroups**

*
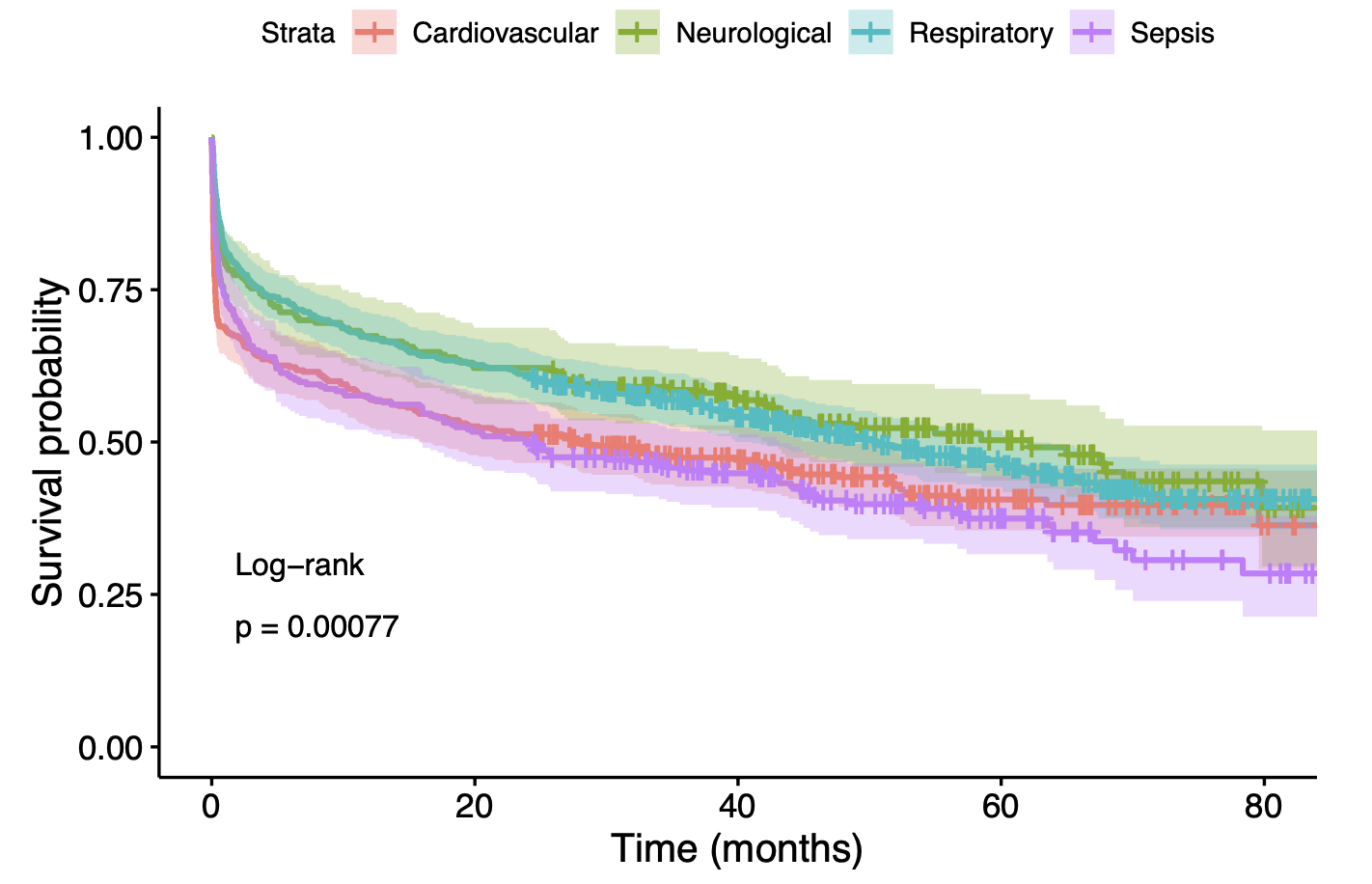
*


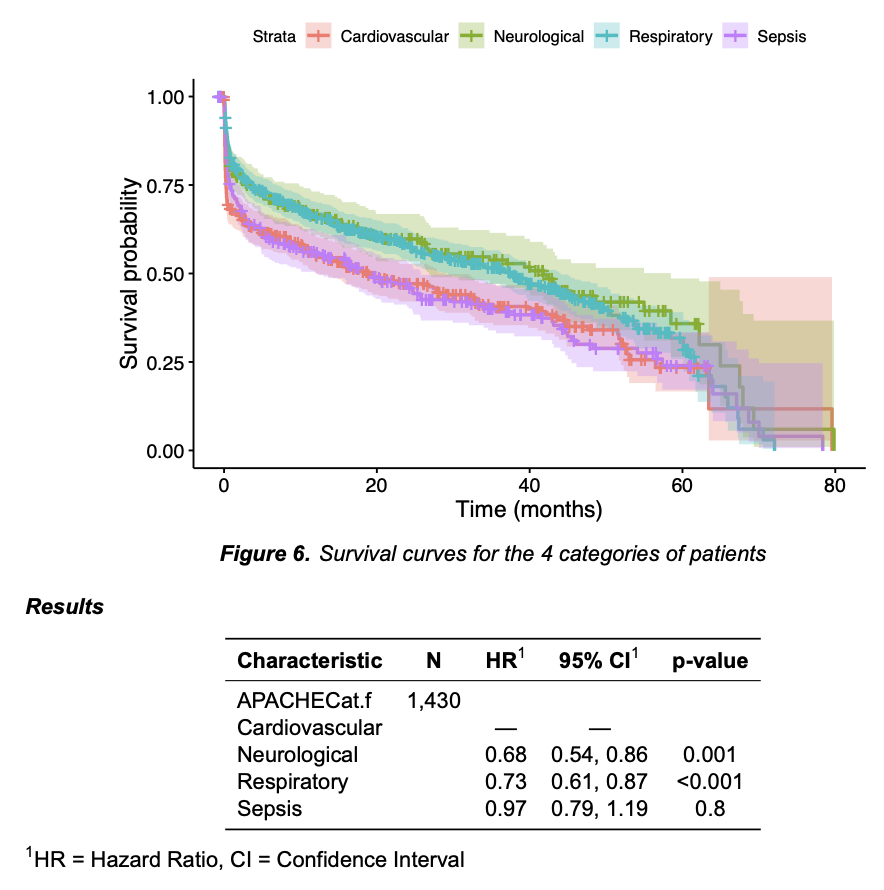


*Supplemental Figure 6. Kaplan-Meier survival curves and Cox hazard regression table across diagnostic subgroups. Higher survival was found in neurological and respiratory subgroups when compared to cardiovascular and sepsis subgroups (HR = 0.68 [0.54-0.86], p=0.001 and HR = 0.72 [0.61-0.86], p<0.001, respectively).*

**Supplemental figure 7. Number of patients in each ‘TAPSE/TRV ratio - TAPSE - TRV’ category across diagnostic groups**

*
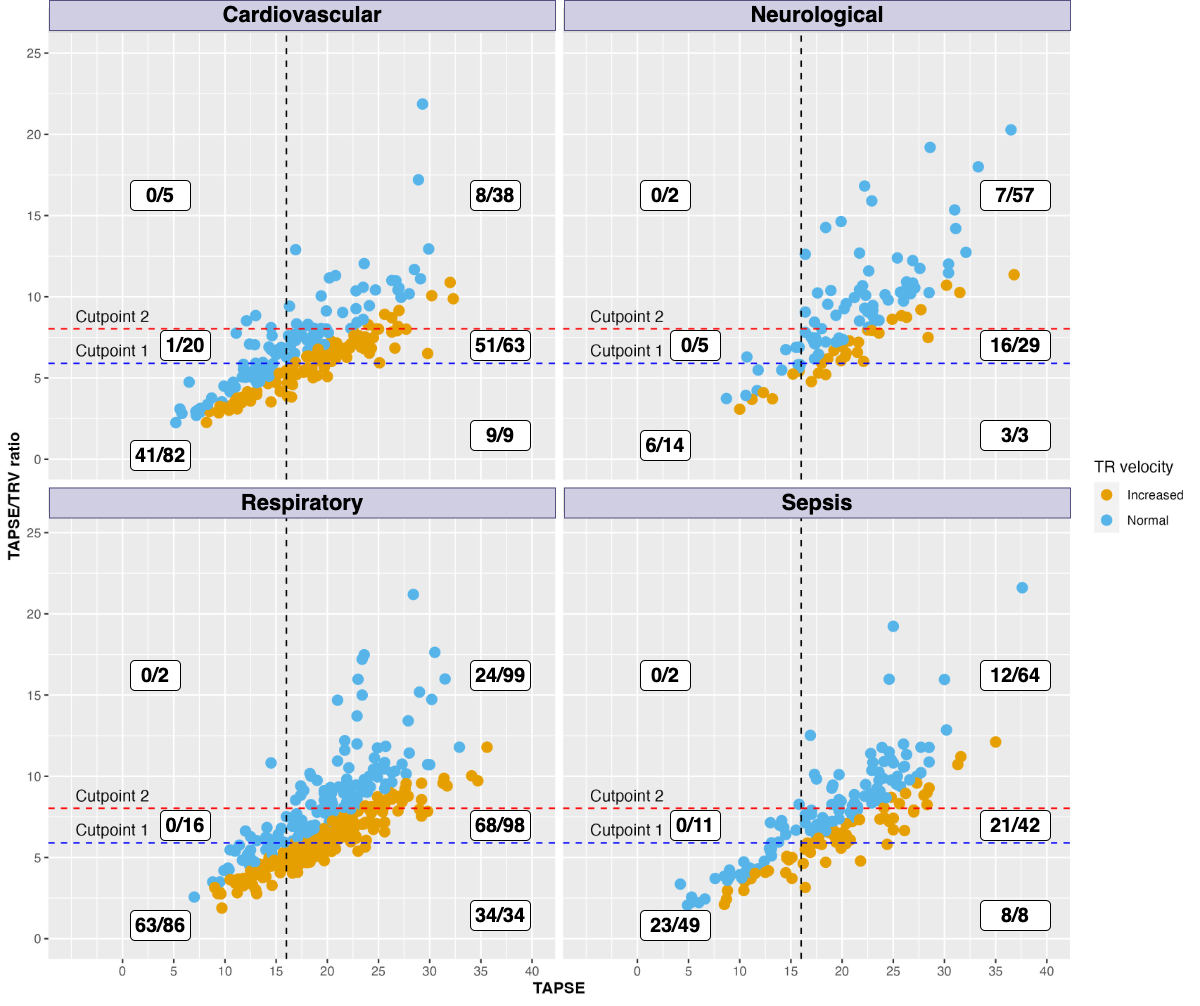
*

*Supplemental figure 7. Blue horizontal dashed line= Cut point 1: 5.9 mm.(m/s)-1 and red horizontal dashed line= cut point 2: 8.03 mm.(m/s)-1. Vertical dashed line = TAPSE <17mm. Fraction represents the number with increased TRV (>2.8 m/s) within that ‘TAPSE/TRV ratio-TAPSE- TRV’ category. If TAPSE/TRV of 8.03 is used as the normal cut point (cut point 2), 11 patients were in the upper left panel with a reduced TAPSE, normal TRV and normal TAPSE/TRV ratio, representing proportion with pseudo-normal TAPSE/TRV ratio with reduced TAPSE group. There were 51 patients in the upper right panel with a high TAPSE, high TRV and normal TAPSE/TRV ratio, representing a pseudo-normal TAPSE/TRV ratio with increased TRV group.*

**Supplemental Table 3. Sequential Cox hazard multivariate analysis**

| Covariate | (1) | (2) | (3) | (4) | (5) | (6) |
| --- | --- | --- | --- | --- | --- | --- |
| Male sex | 1.109 | 1.106 | 1.113 |  |  |  |
|  | [0.809-1.519] | [0.807-1.516] | [0.813-1.524] |  |  |  |
| Invasively ventilated | 1.069 |  |  |  |  |  |
|  | [0.698-1.639] |  |  |  |  |  |
| **Chronic Cardiovascular disease** | **2.302 **** | **2.314 ***** | **2.342 ***** | **2.352 ***** | **2.404 ***** | **2.545 ***** |
|  | **[1.396-3.796]** | **[1.405-3.812]** | **[1.424-3.853]** | **[1.431-3.868]** | **[1.467-3.939]** | **[1.563-4.145]** |
| Chronic Respiratory disease | 1.172 | 1.174 | 1.174 | 1.171 |  |  |
|  | [0.771-1.780] | [0.773-1.783] | [0.773-1.783] | [0.771-1.779] |  |  |
| Normal LV systolic function | 0.777 | 0.782 | 0.793 | 0.778 | 0.781 |  |
|  | [0.540-1.118] | [0.544-1.122] | [0.554-1.135] | [0.546-1.107] | [0.548-1.113] |  |
| Age (decade) | 1.033 | 1.031 |  |  |  |  |
|  | [0.921-1.160] | [0.920-1.157] |  |  |  |  |
| **APACHE 3 score** | **1.033 ***** | **1.034 ***** | **1.034 ***** | **1.034 ***** | **1.034 ***** | **1.034 ***** |
|  | **[1.028-1.039]** | **[1.028-1.039]** | **[1.028-1.039]** | **[1.028-1.039]** | **[1.028-1.039]** | **[1.029-1.039]** |
| **TAPSE/TRV ratio** | 0.940 | 0.940 | **0.938 *** | **0.939 *** | **0.937 *** | **0.927 *** |
|  | [0.882-1.002] | [0.882-1.002] | **[0.881-1.000]** | **[0.881-1.000]** | **[0.880-0.998]** | **[0.872-0.985]** |
| N | 163.000 | 163.000 | 163.000 | 163.000 | 163.000 | 163.000 |
| R2 | 0.157 | 0.157 | 0.157 | 0.157 | 0.156 | 0.155 |
| logLik | -858.355 | -858.403 | -858.544 | -858.766 | -859.032 | -859.952 |
| AIC | 1732.710 | 1730.806 | 1729.089 | 1727.533 | 1726.064 | 1725.904 |
| *** p < 0.001; ** p < 0.01; * p < 0.05. | | | | | | |

*Supplemental Table 3. Sequential Cox hazard multivariate analysis with TAPSE/TRV as a continuous variable and inclusion of age, sex, invasive ventilation status, chronic cardiovascular and respiratory disease, LV systolic function and APACHE 3 score as covariates in a prediction model. After multivariate analysis, chronic cardiovascular co-morbidity, APACHE 3 score and TAPSE/TRV ratio remained independently associated with ICU mortality. HR=Hazard ratio, 95% confidence interval.*
